# Real-World Experience With the Extravascular Implantable Cardioverter Defibrillator Through 1 Year: The Enlighten Post Approval Registry Study

**DOI:** 10.1161/CIRCULATIONAHA.126.080942

**Published:** 2026-09-22

**Authors:** Lucas V.A. Boersma, Anish Amin, Francis Murgatroyd, Maciej Sterliński, Jens Brock Johansen, Karim Saleh, Pamela K. Mason, Jaime Casares, Gregory Engel, David Duncker, Alexander Breitenstein, Theodore Takata, Laurence M. Epstein, Jurren M. van Opstal, Nicolas Clémenty, Paul Friedman, Oliver Monfredi, Marianne Gwechenberger, Nobuhiro Nishii, Christopher Wiggenhorn, Jeff D. Lande, Jennifer Lee, Amy E. Thompson, Thomas R. Holmes, Ian Crozier

**Affiliations:** 1 Department of Cardiology, St. Antonius Hospital, Nieuwegein, Utrecht, the Netherlands (L.V.A.B.).; 2 Department of Cardiology, Amsterdam University Medical Center, Amsterdam, North Holland, the Netherlands (L.V.A.B.).; 3 Heart Health and Cardiac Arrhythmias, Riverside Methodist Hospital, Columbus, OH (A.A.).; 4 Department of Cardiology, King’s College Hospital, London, United Kingdom (F.M.).; 5 I Klinika Zaburzeń Rytmu Serca, Narodowy Instytut Kardiologii, Warszawa, Masovian Voivodeship, Poland (M.S.).; 6 Department of Cardiology, Odense University Hospital, Odense, Syddanmark, Denmark (J.B.J.).; 7 Department of Cardiology and Medical Intensive Care, Kepler University Hospital, Linz, Upper Austria, Austria (K.S.).; 8 Department of Cardiovascular Medicine, University of Virginia School of Medicine 12349 , Charlottesville, VA (P.K.M., O.M.).; 9 Department of Cardiovascular Surgery, Hospital Universitario Reina Sofia 16501 , Cordoba, Cordoba, Spain (J.C.).; 10 Sutter Health, Redwood City, CA (G.E.).; 11 Department of Cardiology and Angiology, Hannover Medical School, Hannover, Lower Saxony, Germany (D.D.).; 12 University Heart Center, University Hospital Zurich, Zurich, Switzerland (A.B.).; 13 Heart and Vascular Specialists, Texas Health Harris Methodist Hospital, Fort Worth, TX (T.T.).; 14 Electrophysiology, Northwell Health 5799 , New Hyde Park, NY (L.M.E.).; 15 Cardiology Department, Medical Spectrum Twente, Enschede, Overijssel, the Netherlands (J.M.v.O.).; 16 Clinique du Millénaire, Montpellier, Occitania, France (N.C.).; 17 Department of Cardiovascular Medicine, Mayo Clinic, Rochester, MN (P.F.).; 18 Division of Cardiology, Medical University of Vienna, Vienna, Austria (M.G.).; 19 Department of Cardiovascular Medicine, Okayama University Graduate School of Medicine, Dentistry, and Pharmaceutical Sciences, Okayama, Chugoku, Japan (N.N.).; 20 Cardiac Rhythm Management, Medtronic Inc 5524 824633 5524 , Mounds View, MN (C.W., J.D.L., J.L., A.E.T., T.R.H.).; 21 Department of Cardiology, Christchurch Hospital 67587 , Christchurch, Canterbury, New Zealand (I.C.).

**Keywords:** cardiac electrophysiology, implantable defibrillator, sudden cardiac death, ventricular fibrillation, ventricular tachycardia

## Abstract

**BACKGROUND:**

The extravascular implantable cardioverter defibrillator (EV-ICD) was developed to provide defibrillation and antitachycardia pacing therapy without the need for intravascular lead placement. Premarket clinical trials reported EV-ICD safety and efficacy through 3 years, but a real-world assessment of the Aurora EV-ICD system, which includes a novel sensing discriminator, is needed. The objective of this analysis was to assess real-world therapy efficacy and safety of the EV-ICD through 1 year.

**METHODS:**

Enlighten: The EV-ICD Post Approval Registry is a prospective, global, real-world registry that included patients who underwent an EV-ICD implantation attempt. The main safety analysis was freedom from all system- or procedure-related major complications through 1 year. Event rates at 1 year were calculated using the Kaplan-Meier method.

**RESULTS:**

EV-ICD system implantation was attempted by 168 implanters in 787 patients (49.1±15.2 years old; 28.7% women; 34.1% secondary prevention). Tunneling and lead placement in the substernal space were achieved in 781 of 787 patients (99.2%), with 758 patients (96.3%) remaining implanted following defibrillation and electrical testing. One-year freedom from major system- or procedure-related complications was 89.1% (95% CI, 86.3%–91.3%). The 1-year rate of first appropriate therapy (shock, antitachycardia pacing, or both) was 7.3% (95% CI, 5.5–9.6). Shock success for discrete spontaneous episodes was 100% (61 of 61), and the generalized estimating equations antitachycardia pacing success rate, to account for multiple episodes per patient, was 74.2% for monomorphic ventricular tachycardia. The 1-year rate of first inappropriate shock was 7.1% (95% CI, 5.4–9.4), with noncardiac oversensing as the main cause (40.5% of inappropriately shocked episodes).

**CONCLUSIONS:**

In the largest prospective worldwide cohort of patients with an EV-ICD to date, both antitachycardia pacing and shock therapy were effective, while the rate of major complications agreed with the premarket experience through 1 year. These data demonstrate that positive outcomes of the EV-ICD are retained in everyday clinical practice in a large, global cohort with an expanded number of implanters.

**REGISTRATION:**

URL: https://www.clinicaltrials.gov; Unique identifier: NCT06048731.

**Clinical Perspective**

**What Is New?**

- The Aurora extravascular implantable cardioverter defibrillator system with a substernal lead shows effective therapy delivery, with 100% discrete shock success and 74.2% adjusted antitachycardia pacing success to terminate ventricular arrhythmias during ambulatory follow-up in a real-world population after commercialization of the device.
- Lead placement success was 99.2%, and the number completing the procedure with a device was 96.3%, while freedom from all major complications was 89.1% through 1 year, none resulting in myocardial injury or intraprocedural surgeon intervention.
- An inappropriate shock rate of 7.1% at 1-year was lower than that observed during the Pivotal trial (9.8%), aided by a reduction in P wave oversensing.

**What Are the Clinical Implications?**

- The extravascular implantable cardioverter defibrillator system provided effective sudden cardiac death prevention over a wide range of etiologies for primary and secondary prevention patients who do not require bradycardia pacing, although the study cohort was somewhat younger and with fewer comorbidities than a standard implantable cardioverter defibrillator population.
- Despite an expansion of implants over a larger number of centers and operators in Enlighten: The EV-ICD Post Approval Registry, the implantation success rates and complication-free rates remain comparable with the Pivotal study experience, demonstrating the therapy has matured and is feasible in everyday clinical practice.
- Future system enhancements are under development for improvement of signal detection and therapy delivery.

Implantable cardioverter defibrillators (ICDs) are a frontline therapy for preventing sudden cardiac death in at-risk patients. Conventional transvenous ICDs have been the gold standard, with decades of clinical data demonstrating their lifesaving potential, but transvenous lead placement can result in serious adverse events such as systemic infection, venous obstruction or injury, cardiac perforation, and thrombosis or complications during chronic lead extraction.^1,2^ The subcutaneous ICD (S-ICD) was developed as an alternative system to avoid vascular lead placement, but its hardware configuration and placement outside of the ribcage preclude anti-tachycardia pacing (ATP) and requires a higher energy output for defibrillation, necessitating a larger device.^3^

The extravascular ICD (EV-ICD), which places a lead under the sternum just above the heart, was designed to provide many of the capabilities of a transvenous ICD (low-energy defibrillation, ATP, pause prevention pacing) while remaining outside of the vascular space. Safety and efficacy outcomes from the EV-ICD Pivotal trial led to worldwide regulatory approvals, including Food and Drug Administration approval in October 2023.^4^ However, more data are needed on the real-world EV-ICD experience outside of a controlled clinical trial setting. Additionally, the postmarket Aurora EV-ICD system differs from the premarket system with the addition of a novel rhythm discriminator (Smart Sense) that is intended to reduce inappropriate shocks due to P wave oversensing, which was the most common contributor to inappropriate shock in the Pivotal trial.^5^ This report will provide the first look at real-world safety and performance outcomes with the Aurora EV-ICD system through 1 year using data from a large, prospective, global registry of patients with an EV-ICD beyond the Pivotal trial.

## Methods

### Data Availability

The data underlying these results will not be made available to other researchers for the purposes of reproducing the results or replicating the procedures.

### Study Design

Enlighten: The EV-ICD Post Approval Registry (NCT06048731) is a global, prospective, observational, multicenter study that is platformed within Medtronic’s long-standing product surveillance registry.^6^ The study protocol was approved by the ethics committees at each participating institution and complies with the guidelines set forth in the Declaration of Helsinki, including obtaining consent from all patients before enrollment and undergoing an implantation attempt with an Aurora EV-ICD system. The postmarket Aurora EV-ICD system includes a novel algorithm (Smart Sense) that was not available on premarket systems. Enlighten also included a cohort of patients from the premarket EV ICD Pilot, Continued Access, and Pivotal trials (NCT03608670 and NCT04060680) who reconsented for continued follow-up in Enlighten. These patients were, however, not included in this particular report, which focuses on patients newly implant with Aurora EV-ICD commercial product. Major complications were adjudicated by an independent clinical events committee, and appropriateness of all treated episodes was adjudicated by an independent episode review committee. All implanting physicians were required to complete a dedicated implanter training program, led by Medtronic, before implanting an EV-ICD system on their own. Of note, the training program was deployed globally upon commercial launch and is independent of whether the implanting physician is part of the Enlighten study. For newly trained implanters, a cardiothoracic surgeon was required to be present for the first 5 cases following training. More details about the Enlighten study can be found in the previously published study design manuscript.^6^

### Overall Safety Analysis

Overall safety was determined by calculating the freedom from all system- or procedure-related major complications through 1 year. All system- or procedure-related major complications that occurred within 1 year were reported in total and stratified by occurrence either ≤30 days or >30 days post implantation.

### Additional Analyses

Additional end points reported in this analysis include a summary of baseline patient characteristics and medical history; characterization of procedure details and outcomes; freedom from chronic, system-related complications; characterization of system revisions and infections; rate of appropriate and inappropriate therapies through 1 year; overall and first-shock success rate for discrete spontaneous ventricular tachycardia (VT)/ventricular fibrillation (VF) episodes; ATP success for monomorphic VT; causes of inappropriate shock; and electrical measurements at implantation (pacing capture threshold, R wave amplitude, impedance) and at 6 months and 1 year (R wave amplitude and impedance). Outcomes were also compared between individuals aged 65 years or older to individuals younger than 65 (see Supplemental Material for more details).

### Definitions

For the purposes of this study and consistent with premarket EV-ICD clinical trials, a system or procedure-related major complication was defined as any adverse event related to the system or procedure that resulted in death, new hospitalization, extension of a current hospitalization by 48 hours or more, system revision (including system explant), or permanent loss of device function due to mechanical or electrical dysfunction of the device. The clinical events committee adjudicated each adverse event to determine its relatedness to the system or procedure, determine whether it was a major complication, and determine whether it was an intraprocedural complication; this included an adjudication of all cardiac deaths and whether they were related to the system or procedure. Procedure success was defined in this analysis as leaving the operating room with a fully functional EV-ICD system in place following electrical testing. Defibrillation testing was not mandatory, and, when performed, there was no required protocol for threshold or step-down testing. Defibrillation testing success was defined as termination of an induced ventricular episode at any energy when performed either during the procedure or the following day. The P and R wave recommendations at implant were defined as an R wave in any vector that is ≥1 mV and (via analyzer or device), P waves either absent/not sensed at ≤0.2 mV, or sensitivity at which P waves no longer sensed ≤0.3 mV with a ratio of R:P wave amplitude ≥10.

### Aurora EV-ICD System Programming

The Aurora EV-ICD system has 3 programmable therapy zones: a nominally “on” VF zone for defibrillation therapy and 2 nominally “off” ATP therapy zones (a fast VT via VF zone and a VT zone). The VF zone has a nominal duration and interval of 30 of 40 beats and 320 ms, respectively. It was recommended during implantation training that 1 or both ATP zones be programmed “on.” If fast VT via the VF zone was programmed “on,” then the detection interval was nominally set to 240 ms. If the VT zone was programmed “on,” then duration and interval were nominally set to 16 beats and 360 ms, respectively. However, individual programming was determined depending on the patient’s needs and clinical history. Sensitivity was established during implantation and was programmed with a safety margin. The recommended sense polarity was the vector in which R and P wave recommendations could be achieved, typically the ring 1–to–ring 2 vector. Nominal settings for other sensing parameters were 150 ms for blank after sense, 360 ms for threshold decay delay, a sensing threshold drop time of 1500 ms, and oversensing prevention feature set to 3.

### Statistical Methods

Continuous variables were reported as either the mean±SD or median with 25th and 75th percentile values for electrical measurements that may be skewed. Categorical variables were reported as the frequency and percentage. All rates through 1 year, including freedom from major system or procedure-related complications, rate of first appropriate and inappropriate therapies, and rate of mortality, were generated with the Kaplan-Meier method. Total numbers of therapies delivered and therapy success rates were reported using data through all follow-ups. ATP success was calculated using both simple proportions and a generalized estimating equations model using logistic regression with an exchangeable correlation structure to account for variability in episode number between patients. Electrical measurement trends over time were evaluated for significance using a linear mixed-effects model with a random intercept for each subject and time treated as quantitative but discrete (0, 1, and 2 assigned for implantation, 6 months, and 12 months, respectively). Primary versus secondary indication for an ICD was determined using patient medical history of a cardiac arrest or a sustained ventricular arrhythmia. Device interrogation (in-office or remote transmissions by an external monitor) to gather device episode data was not performed by all patients but occurred more frequently than clinical follow-up appointments. Thus, the number of patients and the mean and median follow-up duration were calculated separately for device and clinical follow-up. Episode data were considered in those with available device follow-up data, while clinical safety data were considered for all patients with an implant attempt. Statistical methodology for the supplemental age analysis can be found in the Supplemental Methods. Statistical analyses were performed with SAS version 9.4 (SAS Institute, Cary, NC).

## Results

### Enrollment and Baseline Patient Characteristics

From September of 2023 to March of 2025, Enlighten enrolled 805 de novo patients at 112 centers in 23 countries. A total of 47 patients exited the study from enrollment to 12 months of follow-up (see the patient flow diagram in Figure 1). Of those enrolled, 787 underwent an implantation attempt of an Aurora EV-ICD system, performed by 1 of 168 unique implanters. The last implantation procedure occurred in March 2025. Mean clinical follow-up time for patients who completed the procedure with a system in place was 10.5±5.4 months (median, 10.3 months; 0–26.8 months), with 301 patients completing 12 months of clinical follow-up.

**Figure 1.**
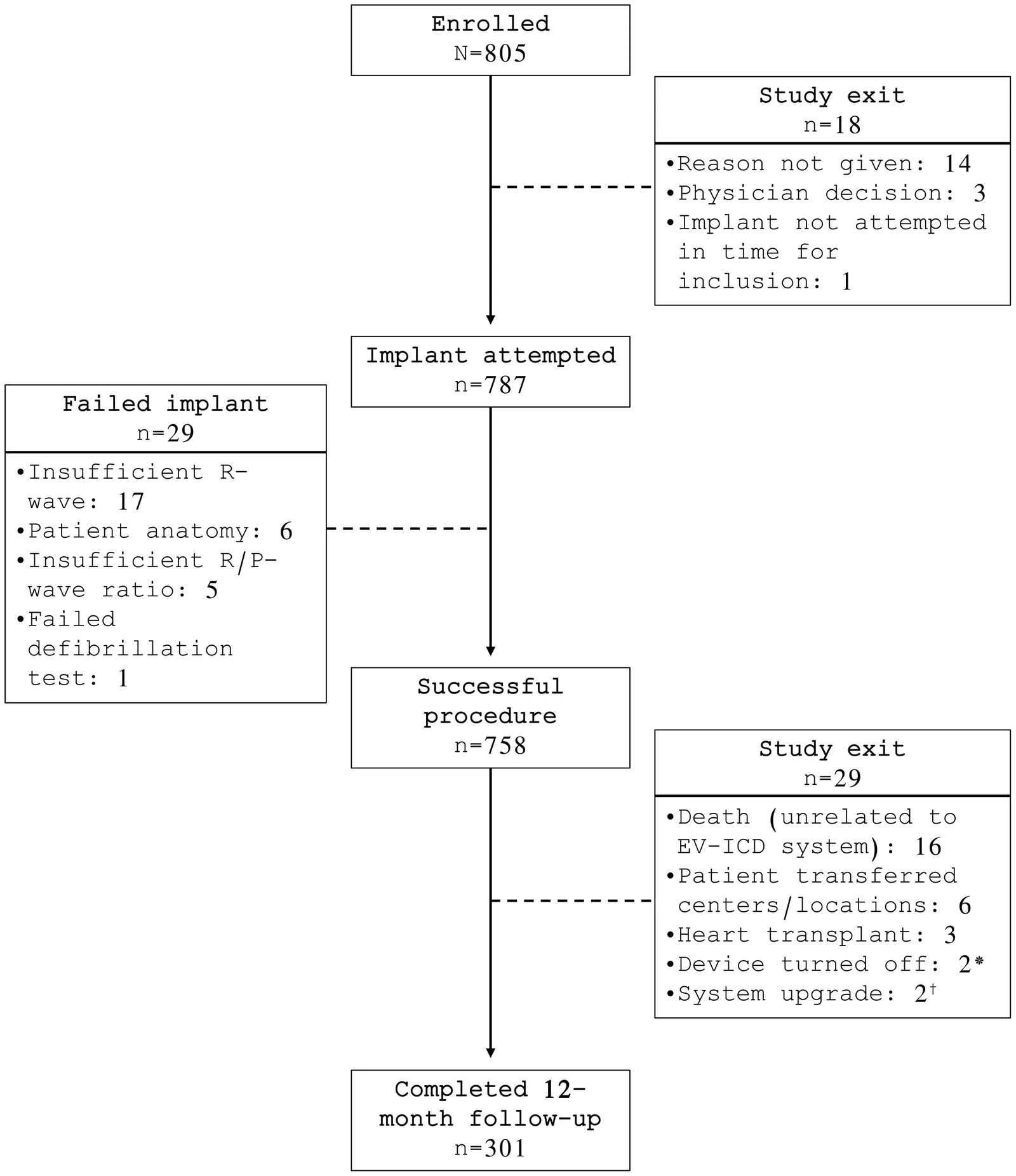
**Patient flow diagram.** The diagram depicts patient flow through the study. A successful procedure was defined as completing the procedure with a system still in place. None of the deaths with a known cause were found to be related to the extravascular implantable cardioverter defibrillator (EV-ICD) system or procedure. *The device was turned off in 2 patients before study exit, 1 following a lead dislodgement and 1 for an end-of-life decision. †One of 2 upgrades resulted in extravascular implantable cardioverter defibrillator system explantation before study exit.

Of patients with an implantation attempt, the mean age was 49.1±15.2 years (136 were ≥65 years old), 226 (28.7%) were women, the mean left ventricular ejection fraction was 44.8±16.0 (n=709), and 268 (34.1%) had a secondary prevention indication for an ICD. A primary or idiopathic electrical disease was documented in 135 (17.2%) patients, 146 (18.6%) presented with hypertrophic cardiomyopathy, 414 (52.6%) had a history of ventricular arrhythmias, and 109 (13.9%) had a history of atrial fibrillation. A full listing of patient characteristics and medical history is provided in Table 1.

**Table 1.**
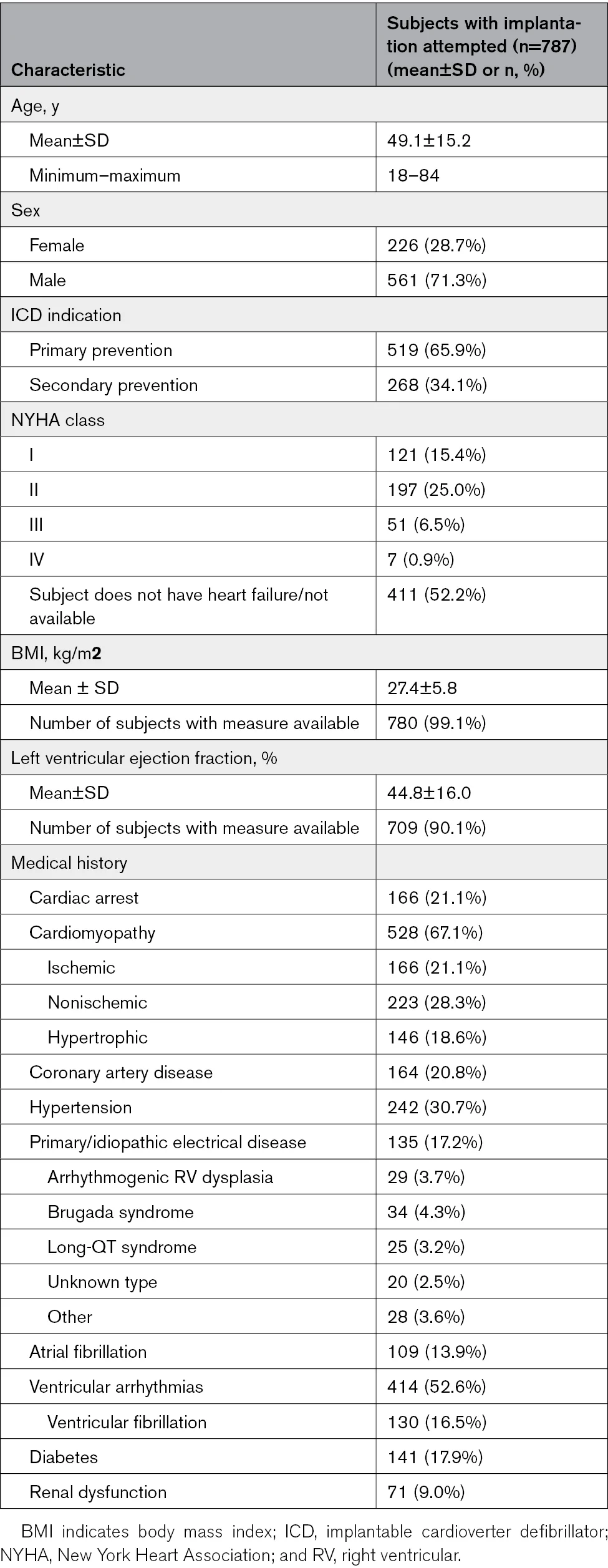
Baseline Patient Characteristics and Medical History

### Procedure and Defibrillation Testing Outcomes

Tunneling and lead placement were successful in 781 of 787 (99.2%) implantation attempts. Following electrical and defibrillation testing, 758 of 787 patients (96.3%) completed the procedure with a system in place. The reasons for not completing the procedure with a system still in place were insufficient R wave amplitude (below 1.0 mV) (n=17), patient anatomy preventing successful tunneling or lead placement (n=6), insufficient R wave–to-–P-wave ratio (n=5), or a failed defibrillation test (n=1). The mean number of reported tunneling attempts for all patients with an implant attempt was 1.3±0.8 tunnels.

Defibrillation testing was recommended but not required and was reported in 716 of 787 patients, of whom 705 (98.9%) were successful at a mean tested energy of 31.2±4.4 J. Six hundred and fifty-two patients were tested at ≤30 J output for at least 1 induced episode, with successful defibrillation in 572 (87.7%) of these patients. Demonstration of the safety margin was at physician discretion, and of the 80 patients not immediately successful with a 10-J safety margin, 75 (93.8%) were successful at higher energy. Fifty-two patients did not undergo defibrillation testing at implantation, the most common reported reasons being a precluding patient condition as determined by the implanting physician (n=17), inability to induce a sustainable ventricular arrhythmia (n=15), and testing planned for a later date (n=8). Troubleshooting to improve the outcome of a defibrillation test was reported for 40 (5.6%) defibrillation tests, with the most common actions being adjustment of the can position (n=25) or checking for or resolving air around the lead (n=7) or in the pocket (n=5). The mean shock impedance during defibrillation testing was 69.5±19.5 Ω. A full list of procedure and defibrillation testing outcomes is reported in Table S1.

### Electrical Measurements

Sensing recommendations for P and R wave amplitude were met in 723 of 740 patients (97.7%), when reported, in patients who completed the procedure with a system in place (see Methods for the definition of sensing recommendations). R wave amplitude measurements at the ring 1–to ring 2 vector remained stable from implantation (2.91±1.61 mV) to 6 months (2.93±1.85 mV) and to 1 year (2.98±1.91 mV) post implantation (*P*=0.27). Lead pacing impedance measured between ring 1 to ring 2 increased from implantation (428.3±187.3 Ω) to 6 months (493.7±134.8 Ω) and to 1 year (509.2±140.4 Ω) post implantation, a trend that was consistent with impedance measurements between ring 1 to coil 2 and high voltage vectors (*P*<0.001 for all). At implantation, the mean pacing capture threshold at ring 1 to coil 2 vector was 5.4±2.1 V (Table 2). Three patients (0.4%) at discharge and 1 patient (0.1%) during follow-up reported ATP being programmed “off” due to pacing sensation during in-office testing.

**Table 2.**
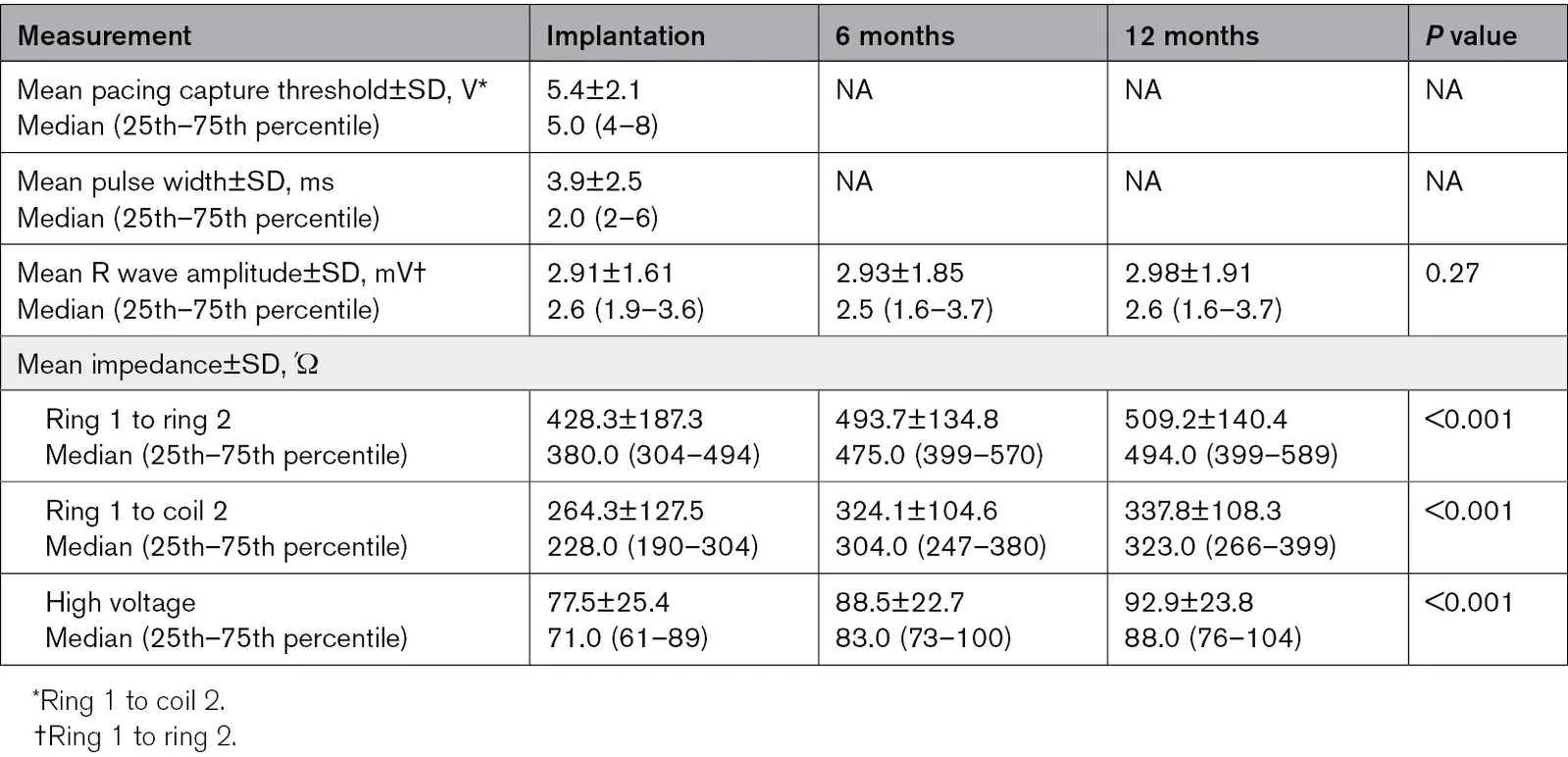
Electrical Measurements Through 1 Year

### Appropriate Therapy

Device programming across treatment zones at the time of the most recent device interrogation is provided in Table S2. Appropriate therapy (shock, ATP, or both) was delivered in 55 of 706 patients with device data for 237 VT/VF episodes through a mean device follow-up time of 12.9±5.6 months (median, 12.8 months). The 1-year Kaplan-Meier–estimated rates of any first appropriate therapy, shock, or ATP were 7.3% (95% CI, 5.5%–9.6%), 5.9% (95% CI, 4.3–8.0), and 3.6% (95% CI, 2.4–5.3), respectively (Figure 2). An appropriate shock successfully terminated 61 of 61 (100%) discrete spontaneous VT/VF episodes. A single shock was successful for 57 (93.4%) discrete spontaneous VT/VF episodes, while 2 episodes required 2 shocks, and 2 episodes required 3 shocks to terminate. Appropriate ATP was delivered for 87 monomorphic VT episodes in 25 patients and was successful for 60 of 87 (69.0%) episodes through all of device follow-up. The generalized estimating equations–estimated ATP success rate, to account for multiple episodes per patient, was 74.2% (95% CI, 57.6–85.9). Shock was potentially avoided in 22 patients for 65 monomorphic VT episodes that only required ATP therapy for termination. No patients who received ambulatory ATP therapy (regardless of appropriateness) had ATP subsequently programmed “off.”

**Figure 2.**
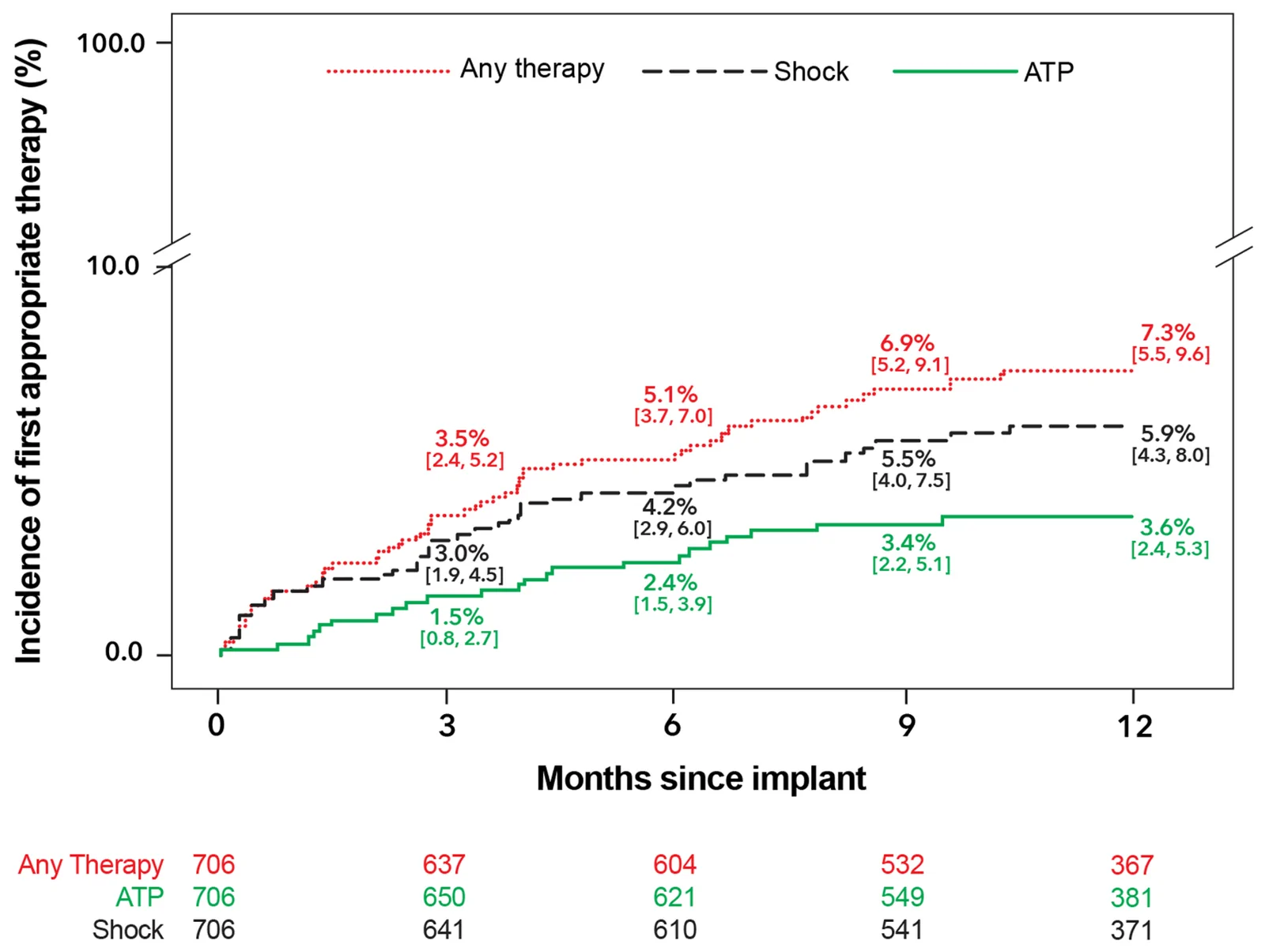
**Rate of first appropriate implantable cardioverter defibrillator therapy through 1 year.** Shown are Kaplan-Meier–estimated rates of first appropriate antitachycardia pacing (ATP) (green), shock (black), or any appropriate therapy (red) through 1 year. This includes 706 patients with available device data. The number at risk for each time point is shown at the bottom in corresponding colors. Numbers in brackets on the graph represent the 95% CI.

### Inappropriate Therapy

Inappropriate shock occurred in 48 of 706 patients with device data for 74 episodes through a mean device follow-up time of 12.9±5.6 months. The 1-year Kaplan-Meier–estimated rate of first inappropriate shock was 7.1% (95% CI, 5.4–9.4) (Figure 3). The causes of inappropriate shock included 30 (40.5%) episodes of noncardiac oversensing (n=20 patients), 23 episodes (31.1%) of P wave oversensing (n=18 patients), 19 episodes (25.7%) of supraventricular rhythm oversensing (n=11 patients), 1 (1.4%) T wave oversensing episode, and 1 (1.4%) nonsustained VT episode (Table S3). Three patients experienced multiple inappropriate shocks for more than 1 of these reasons, 1 of whom experienced inappropriate shocks for both noncardiac and cardiac oversensing. The 1-year rate of first inappropriate shock due to P wave oversensing was 2.8% (95% CI, 1.8–4.4). Inappropriate ATP occurred in 8 patients for 38 episodes through all device follow-up for a 1-year Kaplan-Meier–estimated rate of 1.1% (95% CI, 0.5–2.3). Five of the 38 episodes were also inappropriately shocked. Inappropriate ATP was caused by oversensing of supraventricular tachycardia (36 episodes, 94.7%) or nonsustained VT (2 episodes, 5.3%). The Smart Sense discriminating algorithm was reported as programmed “on” in all but 1 patient, who concurrently had all therapy turned off due to inappropriate shock, as of each patient’s most recent device interrogation. Inappropriate shock resulted in system explantation in 3 (0.4% of the cohort) patients.

**Figure 3.**
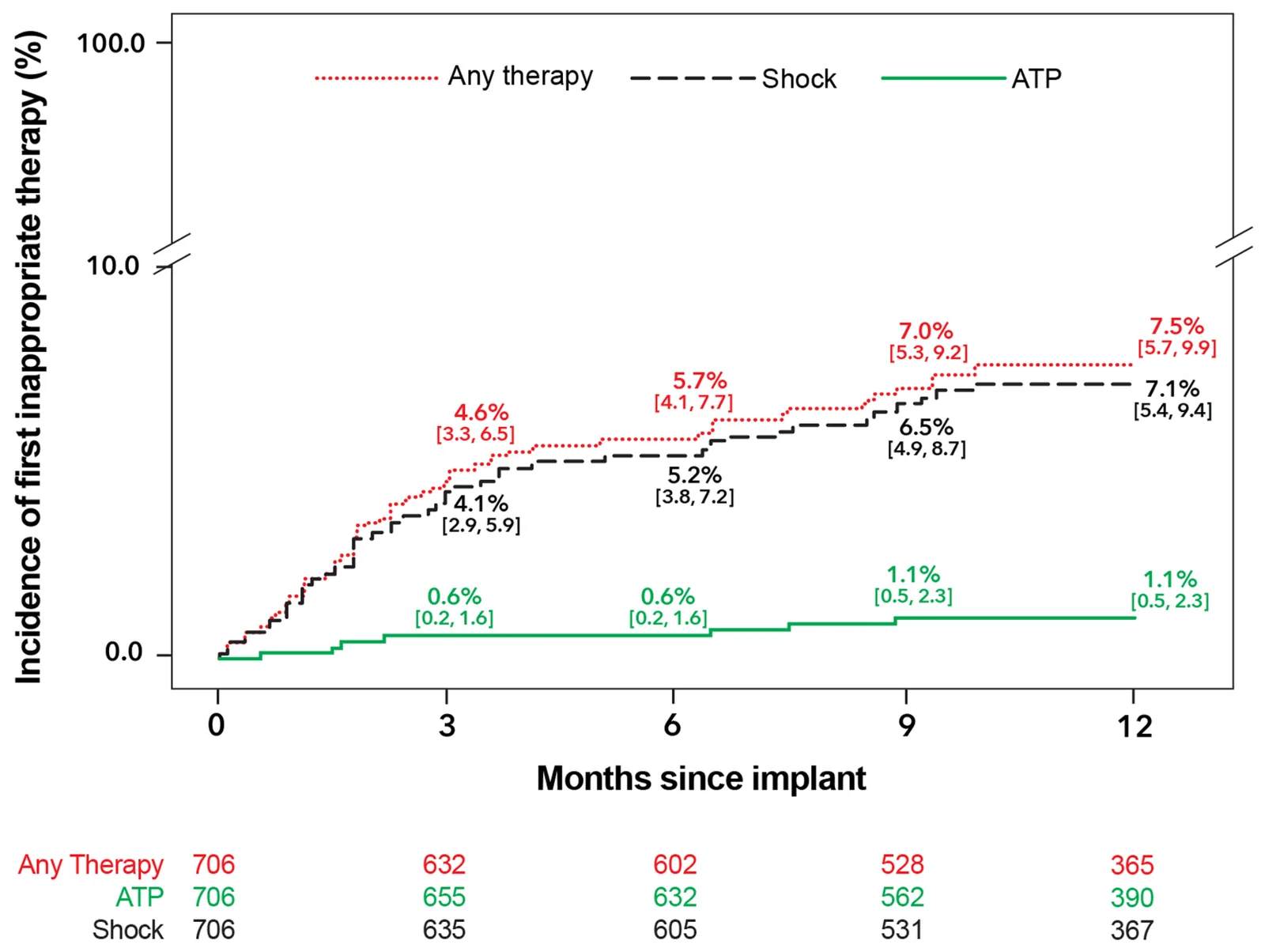
**Rate of first inappropriate implantable cardioverter defibrillator therapy through 1 year.** Shown are Kaplan-Meier–estimated rates of first inappropriate antitachycardia pacing (green), shock (black), or any appropriate therapy (red) through 1 year. The number at risk for each time point is shown at the bottom in corresponding colors. Data include 706 patients with available device data. Numbers in brackets on the graph represent the 95% CI.

### Major Complications

Through 1 year there were 78 major system- or procedure-related complications that occurred in 73 patients, with 44 major complications occurring within 30 days post implantation (Table 3). The Kaplan-Meier estimated freedom from all system- or procedure-related major complications through 1 year was 89.1% (95% CI, 86.3%–91.3%) (Figure 4). Thirty-nine major complications resulted in system revision, 21 of which were a system explant. The most frequently reported major system- or procedure-related complications were lead dislodgment (n=16, 2.0%), infection (17 events; n=15, 1.9%), and inappropriate shock resulting in system revision or hospitalization (n=12, 1.5%). A major system or procedure-related intraprocedural complication occurred in 6 patients. Five were an extension of discharge to either repeat defibrillation testing after a failed test (n=4), of which 2 required device repositioning, or to replace the lead following a lead migration that was noted post procedure (n=1). The sixth was a pneumothorax diagnosed via postprocedure imaging that resolved spontaneously without intervention. There were no reported instances of cardiac injury or intraprocedural surgical intervention. The Enlighten study’s primary end point will assess freedom from chronic, system-related major complications through 5 years (see Supplemental Methods for definition). An update of the primary end point through 1 year is provided in Figure S1 and Table S4).

**Table 3.**
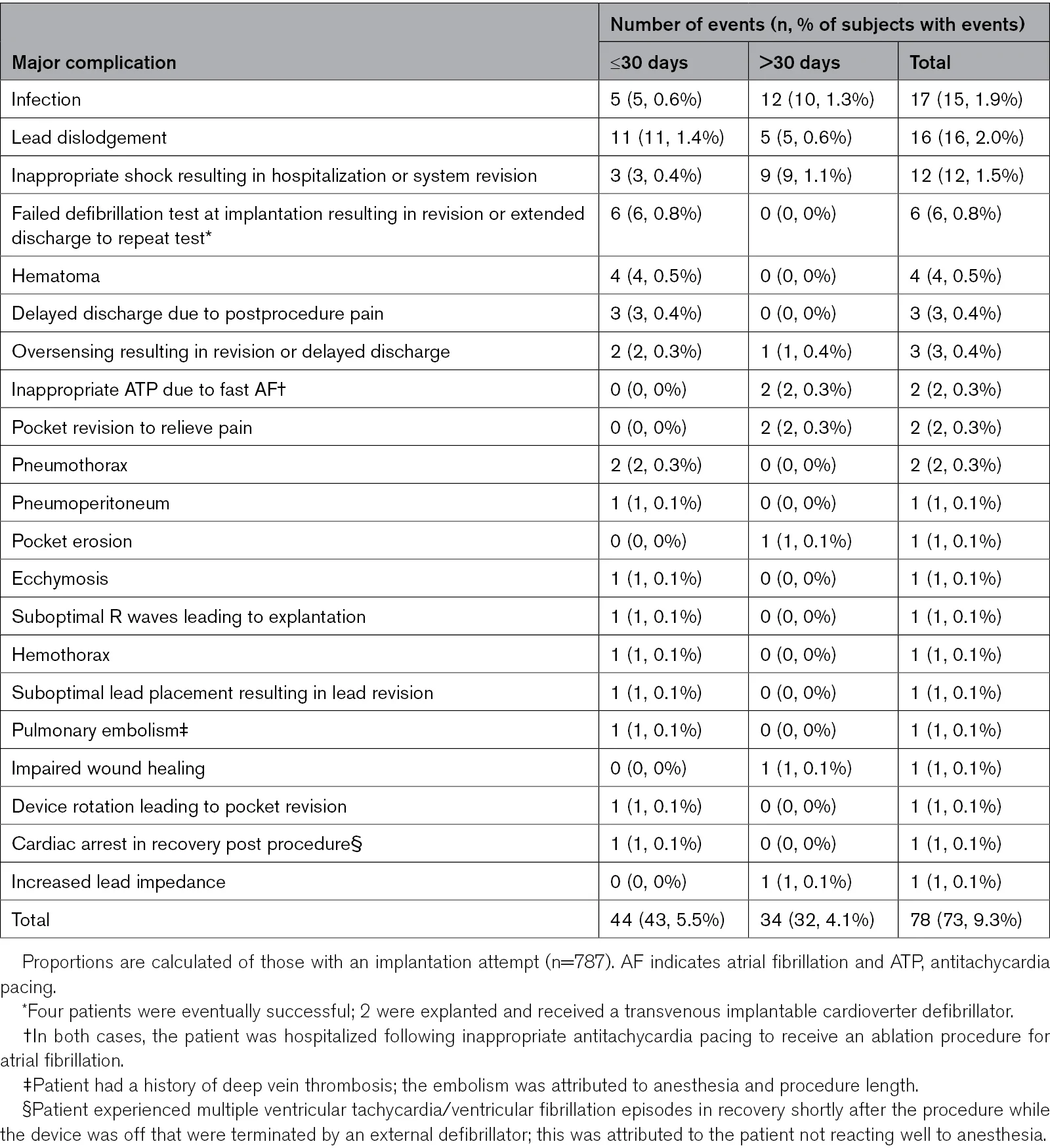
Listing of all system- or procedure-related major complications

**Figure 4.**
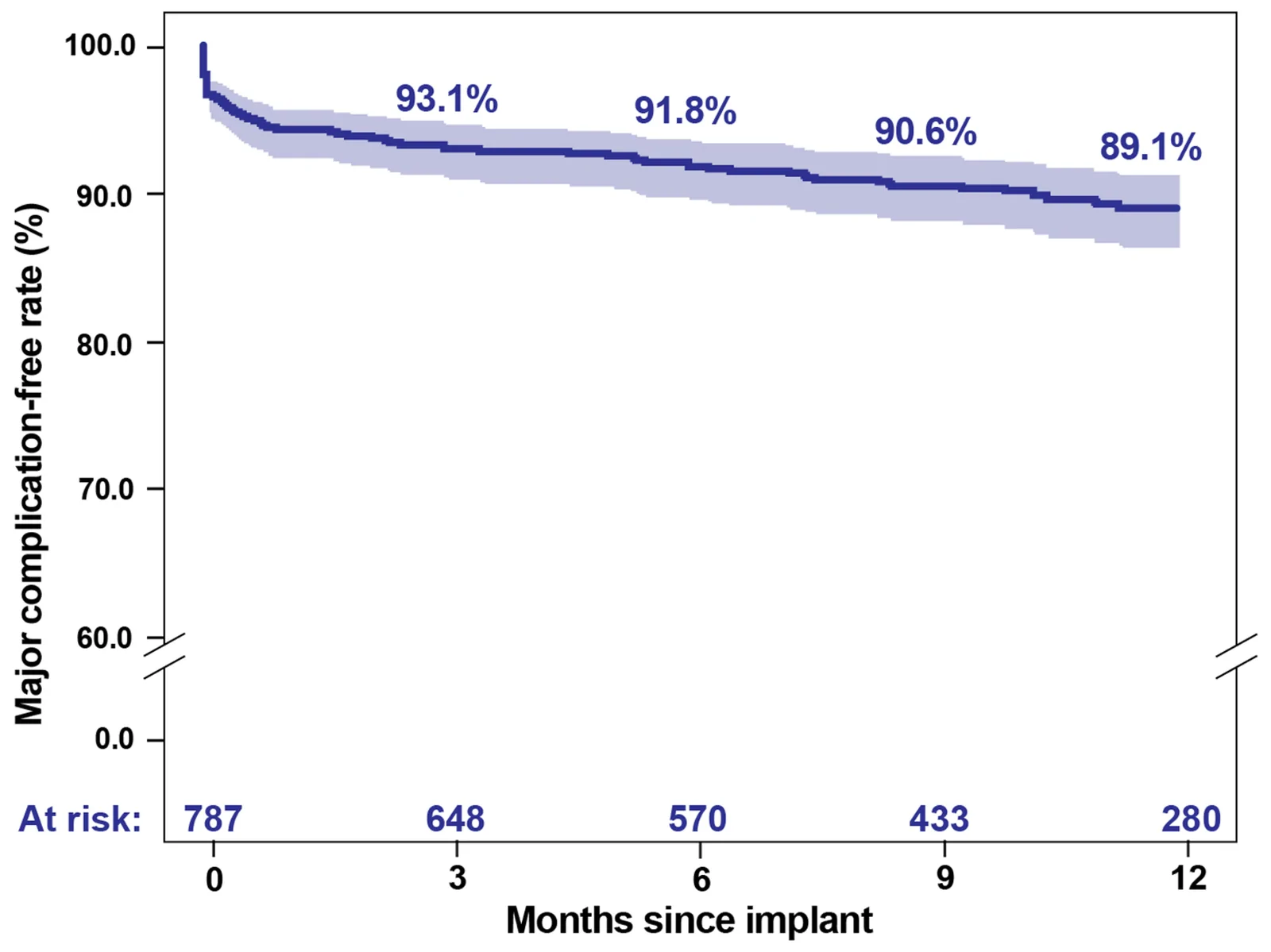
**Freedom from all major system- or procedure-related complications through 1 year.** Shown is Kaplan-Meier–estimated freedom from all system- or procedure-related major complications through 1 year for 787 patients with an implantation attempt. The number at risk for each time point is shown at the bottom of the graph in blue. The blue-shaded region represents the 95% CI.

### Infections

All infections related to the system or procedure that required system revision, explant, or a new hospitalization were defined as major complications. Through 1 year of follow-up, there were 17 such infections in 15 patients (1.9%) identified 5 to 348 days post implantation. Nine of the infections were associated with the lateral device pocket or incision (n=9 patients), 7 with the subxiphoid incision (n=5 patients), and 1 with both (n=1). Eleven patients underwent surgical revision or explant due to infection (1.4% of the cohort): 1 required a pocket revision, 1 underwent 3 separate revisions of the subxiphoid incision for recurrent infection, and 9 patients underwent a complete system removal. Fifteen of the 17 major complication infections were resolved without sequelae, while 2 that resulted in system removal were still undergoing treatment at the time of patient exit. Fifteen additional infections were adjudicated as minor (n=1) or observations (n=14); all 15 resolved following conservative treatment, and none were included as major complications. A TYRX antibacterial envelope was used in 222 of 787 (28.2%) patients. There were no cases of system- or procedure-related infection that progressed to sepsis, mediastinitis, or endocarditis among patients with an implantation attempt.

### System Revisions

A revision or removal of the lead or device was performed in 43 (5.7%) patients through 1 year (0–342 days post implantation). The types of revision included complete system removal (n=25), repositioning of 1 or more system components (n=10), and lead removal and subsequent replacement of the lead (n=8) (Table S5). Reasons for a complete system removal were infection (n=9), lead dislodgement (n=5), inappropriate shock (n=3), heart transplantation (n=3), failed defibrillation test (n=2), device erosion (n=1), upgrade to a cardiac resynchronization therapy defibrillator (n=1), and insufficient R wave (n=1). The system explants due to either heart transplantation or upgrade to a cardiac resynchronization therapy defibrillator (4 of 25 explants) were not related to the EV-ICD system or procedure and were not considered major complications. Following complete removal of the EV-ICD system, 7 patients were known to have received a new system as follows: a transvenous ICD (n=5), S-ICD (n=1), or cardiac resynchronization therapy defibrillator (n=1). There were 298 of 787 (37.9%) patients with an implantation attempt who completed 1 year of clinical follow-up and did not require a system revision. There were 391 active patients who had not yet completed 1 year of clinical follow-up and did not require a system revision.

### Deaths

There were 16 deaths that occurred through 1 year, with none being related to the EV-ICD system or procedure. Relatedness to the system or procedure could not be determined for 3 deaths due to a lack of information about the cause of death (Table S6). The Kaplan-Meier–estimated freedom from all-cause mortality through 1 year was 97.1% (95% CI, 95.2–98.2).

### Assessment of Outcomes by Age

Safety and efficacy were compared between Enlighten patients aged 65 or older (n=136) and those younger than 65 (n=651) (Table S7). There was no significant difference in implant success between groups (*P*=0.074) (Table S8). Freedom from all major system- or procedure-related major complications was consistent between groups (*P*=0.81), and appropriate and inappropriate therapy rates, though numerically higher in patients <65 years old, were not significantly different between groups through 1 year (Figures S3 and S4). Full details are provided in the Supplemental Results.

## Discussion

These results represent the largest cohort of patients with an EV-ICD to date and the first real-world assessment of the Aurora EV-ICD system through extended follow-up of 1 year. The Enlighten study included an expanded number of implanters compared with the premarket studies, many of whom were new to the implantation procedure (140 of 168 were unique postmarket implanters). Patients in the present cohort were younger than in most standard ICD cohorts (49.1±15.2 years old) with a variety of etiologies, and many had a history of ventricular arrhythmias, translating to a substantial proportion with a secondary prevention ICD indication (34.1%). In real-world clinical practice, tunneling and lead placement were achieved in a majority of patients (99.2%), with 96.3% remaining implanted following defibrillation and electrical testing. Freedom from all major system- or procedure-related complications was 89.1% at 1 year, and both shock and ATP therapy were effective at terminating spontaneous VT/VF episodes. The results help to demonstrate the performance of the EV-ICD system in a real-world clinical setting.

### Periprocedural Outcomes

Periprocedural results from the Enlighten study were reported for the first 228 patients with an implantation attempt and shown to be in line with a pooled cohort of premarket patients.^7^ Those initial procedural data have remained consistent in the full cohort of 787 patients reported here. In the full cohort, 96.3% had a successful implantation procedure, with insufficient R wave amplitude or high P waves being the predominant reason for failure (22 failed procedures, 2.8% of all attempts). Several factors may influence R wave amplitude at implantation, including lead position, patient anatomy, or introduction of air around the lead during tunneling. In published cases of air ingress, R waves were restored to optimal levels the following day once air was no longer present.^8,9^ In Enlighten, it is unclear how many failed implantation attempts due to insufficient R waves would have improved if reassessed the following day. Therefore, implantation failure due to low R wave amplitude in a small subset of patients is a current limitation of the EV-ICD system, and future work will be needed to identify measures to improve R wave sensing in patients who present with suboptimal sensing at implantation or during follow-up. While R wave amplitude remained through 1 year, impedance values increased over time before leveling off, which was also observed in the Pivotal trial and should be expected with the EV-ICD system due to tissue changes post procedure in the retrosternal space, though the exact mechanism has not yet been studied. Gradual changes in high voltage impedance remaining below 250 Ω are generally considered clinically irrelevant with the EV-ICD.

Defibrillation testing during the procedure or next day was successful in 98.9% of patients. While most patients were tested with a 10-J safety margin, 5.8% were only tested at a maximum energy of 40 J. This may reflect physicians’ preference for a maximum‑energy test to avoid prolonging the procedure and reducing the risk of cardiac stress from repeated inductions and shocks. Also, implanters may feel confident about outcomes of defibrillation testing obtained in transvenous ICD studies. The procedural outcomes from this study, with 140 of 168 implanters being newly trained in the postmarket phase, highlight the success of an effective implantation training program and the acute performance of EV-ICDs in the real world.

### Real-World safety

Through 1 year, the safety outcomes of the EV-ICD system in real-world clinical practice have followed the results from the premarket clinical studies. An 89.1% freedom from all major system- or procedure-related complications through 1 year is comparable to the Pivotal trial rate through 1 year (91.9%). It is worth noting that Enlighten patients were younger with fewer comorbidities than patients in the Pivotal study and even more so when compared with transvenous ICD cohorts. Though it is difficult to compare complication rates with other types of ICD systems due to differences in patient demographics and how complications are defined between studies, the rate in Enlighten is consistent with those shown for other ICD systems.^10,11^ Lead dislodgement was the most common major complication (2.0% within 12 months), although the incidence is lower than in the Pivotal study (2.8% within 6 months) and is typically also one of the most common complications reported for transvenous ICDs, especially in younger, more active patients.^4,12^ Major intraprocedural complications were noted in 6 patients in Enlighten while none occurred in Pivotal. Importantly, all 6 resolved, with only 1 resulting in intervention for a lead migration that necessitated lead replacement, while none caused any permanent damage to the heart, vasculature, or lungs.

One value proposition of an extravascular system is the potential elimination of serious complications associated with implanting leads that reside indefinitely within the veins and heart, particularly the risk of systemic infection. A major system or procedure-related infection occurred in 15 (1.9%) patients, with 9 (1.1%) resulting in system explantation. All other major infections were managed with routine care, and antibiotics alone were administered in 4 cases. In every EV-ICD study to date, including the current Enlighten report, infections have remained localized to the pocket or incision, with none progressing to sepsis, endocarditis, or mediastinitis, and explantation procedures and extractions were uneventful. However, the younger and overall lower risk profile of the EV-ICD population compared with a standard ICD patient may impact the infection rate and severity. There were 25 of 758 (3.3%) EV-ICD systems explanted within 1 year in patients with a successful procedure. This rate is comparable with what was reported in the S-ICD postapproval study through 1 year (59 of 1637, 3.9%).^13^

### Therapy Effectiveness

Substernal lead placement with the EV-ICD system allows for defibrillation at an energy similar to transvenous ICD and ATP therapy. The 1-year rate of appropriate therapy is higher in Enlighten than that observed in the Pivotal trial (7.3% versus 4.7%), likely due to the larger proportion of Enlighten patients with a secondary prevention indication for an ICD (34.1% versus 18.0%). Shock success in the Pivotal trial was 100% for 27 discrete spontaneous episodes through 3 years.^14^ Thus far, Enlighten has also shown 100% shock success for 61 discrete spontaneous episodes, and the first-shock success of 93.4% is in line with that reported for transvenous (90%) and S-ICDs (91%).^13,15^ This result demonstrates the ability of the EV-ICD to successfully defibrillate the heart during ambulatory activities. While defibrillation testing is currently recommended for the EV-ICD, the first-shock success rate is one of several important reassurances to consider in cases where defibrillation testing cannot be performed. Nevertheless, regardless of whether defibrillation testing is performed, proper implant technique to optimize lead and device position is the first step in ensuring success for spontaneous shocks and may likely underscore the first shock success rate.

In addition to effective shock therapy, ATP was also effective, with a 69.0% success rate (74.2% when generalized estimating equations adjusted), which led to potential avoidance of shock in 22 patients for 65 terminated episodes only treated with ATP. The success rate is consistent with the generalized estimating equations–adjusted ATP success rate in Pivotal of 72.5%^14^ and that reported for transvenous systems.^16,17^ While ATP provides benefits, particularly through avoiding shocks, which can deteriorate patient quality of life,^18^ ATP can accelerate the arrhythmia or delay potential lifesaving therapy if unsuccessful. The rate of ATP acceleration for transvenous ICDs has ranged from 2% to 5%, and as yet there is no indication that this rate is any different for EV-ICD, although a formal analysis is needed.^17,19^ Pacing capture thresholds with EV-ICDs are higher than what is seen with transvenous ICDs, which could make ATP more perceptible. While ATP is nominally “off” in the EV-ICD device, it was programmed “on” in 88% of patients, per physician discretion, highlighting the perceived patient benefit. Four (0.5%) patients had ATP programmed “off” at any point due to pacing sensation during in-office testing, but no patient who received any appropriate or inappropriate ambulatory ATP therapy subsequently had ATP programmed “off.”

### Inappropriate Shock

While the EV-ICD has exhibited a strong safety profile with robust therapy success, inappropriate shocks are higher than what is typically shown for transvenous ICDs, with P wave oversensing as the predominant cause in the Pivotal trial.^14,20^ To help address this, a novel P wave discriminator (Smart Sense) was added to the postmarket Aurora EV-ICD to mitigate atrial oversensing. In a previous analysis, modeling of Pivotal trial episodes showed a 29% reduction in the inappropriate shock rate with use of Smart Sense.^5^ In real-world clinical practice with Smart Sense, the Enlighten study demonstrated an inappropriate shock rate of 7.1% through 1 year, a 28% reduction compared with the 9.8% rate at 1 year reported in the Pivotal trial.^14^ The Kaplan-Meier rate of inappropriate shock due to P wave oversensing was 2.8% in Enlighten versus 3.4% in Pivotal.^14^ A deeper analysis of Smart Sense performance, including rate of appropriately withheld shocks, is needed to assess the true impact of the algorithm on inappropriate shock reduction. There are probably several other factors contributing to the decrease in inappropriate shock, including physician experience, patient selection, programming mitigations, and improved implantation technique to minimize P waves. A 1-year inappropriate shock rate of 7.1% is in line with that reported for S-ICDs in postapproval and registry studies ranging from 6.7% to 8.7%,^10,13^ while 1-year rates with the latest S-ICD model have been as low as 2.4% in the UNTOUCHED study of patients with primary prevention of low left ventricular ejection fraction and as high as 8.5% in the MODULAR ATP Trial with a mixed cohort of primary and secondary prevention patients.^21,22^ Transvenous single-chamber ICD rates from landmark trials with modern programming have ranged from around 5% to as low as 2.5%.^16,23^

Added implanter experience and algorithm updates may reduce inappropriate shocks with the EV-ICD over time, but new data will be needed to assess the true impact of each on inappropriate shock reduction in clinical practice. In addition to novel device algorithms such as Smart Sense, implantation technique and device programming are currently highly important in mitigating inappropriate shock. Properly securing the lead and meeting P and R wave recommendations can reduce the risk of lead dislodgement and P wave oversensing, respectively. The EV-ICD has several programming features that can be adjusted to mitigate de novo or subsequent inappropriate shocks. These include changing the sensing vector, increasing the time to detection, adjusting the sensitivity level, or adjusting the oversense prevention setting. In the Pivotal trial, 73.2% of patients who received an inappropriate shock and who were managed without a system revision were free from a subsequent inappropriate shock through a mean follow-up of 19 months after interrogation, with a majority of those patients’ devices being reprogrammed.^14^ An evaluation of the impact of patient characteristics, implantation outcomes, and EV-ICD programming changes on inappropriate shocks in Enlighten is forthcoming.

### Implications of Patient Age

Physicians utilized EV-ICDs in young patients (49.1±15.2 year average age in Enlighten), presumably reflecting physicians’ perception of the importance of preserving the vasculature and avoiding serious complications that could arise with very long transvenous lead dwell times. Therefore, data on the safety and performance of EV-ICDs by patient age is warranted. In the current cohort, there was no difference in the rate of major system- or procedure-related complications or in therapy efficacy between older and younger patients. These preliminary results suggest that the EV-ICD is performing equally across all age groups in Enlighten, but further evaluation is needed to assess outcomes, particularly safety, in a cohort of older patients with additional comorbidities that are typical of transvenous ICD populations.

### Limitations

Several limitations must be considered when evaluating the results of this analysis. This was a single-arm study with no randomization to compare with other types of ICDs or with guideline-directed medical therapy. The absence of prespecified protocols in a real-world registry makes it difficult to compare certain outcomes, such as defibrillation testing success, with the preclinical Pivotal trial, which had a more structured approach. Device data were not available for all patients, but major safety datapoints were assessed for all patients with an implantation attempt. While the median device follow-up was 12.8 months, the median clinical follow-up was somewhat less (10.3 months), with fewer than half of patients having had 12 months of clinical follow-up reported, so some outcomes may shift slightly with the full cohort. This analysis was limited to 1 year, and longer-term real-world data are needed, particularly for experience with chronic lead extraction and long-term management of complications. Lead removal data from the premarket EV-ICD studies and guidance for approaching EV-ICD lead extraction have been published previously.^24^ The Enlighten cohort is younger with fewer comorbidities and with a higher proportion having a secondary prevention indication than what is typically seen for transvenous ICD patients; while we provide a preliminary analysis of patients older than 65, more data will be needed to assess EV-ICD outcomes in a comparable transvenous population.

### Conclusions

Real-world clinical evidence from a large, global population of relatively young patients with an Aurora EV-ICD system demonstrated effective defibrillation and ATP therapy through 1 year with freedom from complications in line with premarket studies. The EV-ICD system provides a clinically impactful therapeutic option for sudden cardiac death prevention for both primary and secondary prevention patients who do not require bradycardia pacing.

## Article Information

### Acknowledgments

The authors thank Cory Jensen, MS, of Medtronic, for help with statistical analysis and statistical validation.

### Disclosures

Dr Boersma reports consulting fees and grant support from Medtronic, Boston Scientific, and Philips, paid to the cardiology department at his institution. Dr Amin reports an institutional grant from Biosense Webster; consulting fees from Medtronic, Boston Scientific, Biosense Webster, Atricure, and Philips; honoraria from Medtronic, Boston Scientific, Biosense Webster, Atricure, and Philips; travel support from Boston Scientific, Medtronic, and Philips; and advisory board participation at Boston Scientific, Medtronic, Atricure, and Philips. Dr Murgatroyd reports consulting fees from Medtronic for advisory board and steering committee participation, consulting fees from Boston Scientific for steering committee participation, and speaker fees from Medtronic. Dr Sterliński reports investigational, lecture, proctoring or consultancy fees from Abbott, Biotronik, Boston Scientific, Medtronic, Polpharma, and Zoll. Dr Johansen reports honoraria paid to his institution from Merit Medical and education course, and advisory board participation at Medtronic and Biotronik. Dr Saleh reports lecture, proctoring, and consultancy fees from Abbott, Biotronik, Medtronic, Merit Medical, Microport, and Boston Scientific. Dr Mason reports consulting for Medtronic, Boston Scientific, and Cook as well as honoraria from Medtronic and Cook. Dr Engel reports consulting fees from Medtronic. Dr Duncker reports lecture honoraria, travel grants, or a fellowship grant from Abbott, Astra Zeneca, Bayer, Biotronik, Boehringer Ingelheim, Boston Scientific, Bristol Myers Squibb, Medtronic, Microport, Pfizer, Sanofi, Zoll, and Avid. Dr Breitenstein reports receiving consulting or speaker fees from Abbott, Bayer Healthcare, Biosense Webster, Biotronik, Boston Scientific, Bristol-Myers Squibb, Cook Medical, Daiichi Sankyo, Medtronic, Pfizer, Philips, and Zoll. Dr Takata reports research support and consulting fees from Medtronic. Dr Epstein reports consulting for Medtronic and personal fees from Philips for consulting. Dr Clémenty reports consulting fees, honoraria, and travel support from Medtronic and advisory board participation at Medtronic. Dr Friedman reports licenses and patents with Anumana, Eko Health, Alive Cor, and Marani Health; advisory board participation at Anumana; a leadership position with xAI.health and MediCool; steering committee participation and advisory group participation at Medtronic and Boston Scientific; and funds paid to his institution and stock from MediCool, Marani Health, and xAI.health. Dr Monfredi serves as a consultant for Medtronic, Philips, and Boston Scientific. Dr Gwechenberger reports personal fees (lectures, advisory boards, and research grants) and travel grants from Daiichi Sankyo, Boehringer Ingelheim, Bayer, Abbott, Biotronik, Boston, Medtronic, and Sorin. Dr Nishii reports consulting for Medtronic and affiliation with an endowed department by Medtronic Japan. Dr Wiggenhorn, Dr Lande, Dr Lee, Dr Thompson, and Dr Holmes report employment at Medtronic. Dr Crozier reports consulting for and has received research support and fellowship support from Medtronic and grants from Boston Scientific

### Supplemental Material

Supplemental Methods

Supplemental Results

Figures S1–S2

Tables S1–S8

## Supplementary Material
