# Supplementary material for: Real-World Experience With the Extravascular Implantable Cardioverter Defibrillator Through 1 Year: The Enlighten Post Approval Registry Study
Source: Circulation. 2026 Sep 22;154(19):1925–38. doi: 10.1161/CIRCULATIONAHA.126.080942 (PMC13742346; doi:10.1161/CIRCULATIONAHA.126.080942)
Supplement: Supplementary file 1 [file cir-154-1925-s001.pdf]

Supplemental Methods

Supplemental statistical analyses

Patients were placed into one of two groups based on their age at enrollment: an older cohort of those aged 65 or more, and a younger cohort of those less than 65 years old. Baseline characteristics and procedure details were compared using Fisher's exact test or a 2-sample t-test. A log-rank test was used to compare event rates through one year between groups. All other methods and definitions followed what was outlined in the main methods section. The regulatory required endpoint of the Enlighten Study is freedom from chronic (occurring > 30 days post-implantation) system-related major complications through five years, excluding infections. An early look at this endpoint through one year is provided in the supplemental results below.

Supplemental Results

One-year snapshot of the primary endpoint

The Enlighten primary endpoint will officially be assessed at five years. The one-year freedom from chronic (occurring >30 days post-implantation) system-related complications, excluding infections, was 95.8% (95% CI: 93.6, 97.2) (**Supplemental Figure 1**). This is comparable to the Pivotal trial rate of 97.3% for this same endpoint. The most common major complications that met this endpoint through one year are listed in **Supplemental Table 4**.

One-year outcomes by age

There were 136 patients aged 65 or more (mean age: 70.4 ± 4.5 years; mean follow-up 9.7 ± 6.4 months; mean device follow-up: 13.1 ± 5.6 months) and 651 patients younger than 65 (mean age: 44.6±12.6 years; mean follow-up 10.1 ± 5.6 months; mean device follow-up: 12.8 ± 5.6 months) with an EV-ICD implantation attempt (**Supplemental Table 7**). Those 65 or older had a lower proportion of women (20.6% versus 30.4% female), a lower mean left ventricular ejection fraction (41.2 ± 15.9% versus 45.5 ± 16.0%), higher proportion with a secondary prevention indication for an implantable cardioverter defibrillator (ICD) (37.5% versus 33.3%), and more likely to have a history of atrial fibrillation (28.7% versus 10.8%).

Tunneling and lead placement success rates were similar between older (100%) and younger patients (99.1%) (P=0.60). Likewise, defibrillation testing success was similar between patients 65 or older (100%) and those younger than 65 (98.7%) (P=0.62). Mean procedure time from first incision to final suture was 77.8 ± 31.3 minutes in those 65 or older compared to 81.7 ± 39.3 in patients younger than 65 (P=0.25). There was no difference in the mean number of tunneling attempts between groups (P=0.18). Final implant R-wave amplitude measurement was

3.0 ± 1.7 mV in the younger than 65 cohort compared to 2.3 ± 1.1 mV in patients 65 or older (P<0.001) (**Supplemental Table 8**).

There was no difference in the freedom from all system- and/or procedure-related major complications through one year between groups (P=0.81) (**Supplemental Figure 2**). Of 17 major system- and/or procedure-related infections that occurred, 15 (2.0%) were in those younger than 65 and two (1.5%) among those 65 or older through 12 months. All 15 infections adjudicated as minor (n=1) or observations (n=14) occurred in the cohort younger than 65 with none in the patients 65 or older.

Appropriate therapy rate through one year was numerically higher in patients younger than 65 compared to patients 65 or older but not significant (7.9% vs 4.1%; P=0.24) (**Supplemental Figure 3**). Similarly, the one-year inappropriate shock rate was numerically higher in those younger than 65, but not significantly different compared to those 65 or older (7.5% vs 4.8%; P=0.27) (**Supplemental Figure 4**). Antitachycardia pacing (ATP) was successful for 69.2% (9/13) of ATP-treated episodes in patients 65 or older and 68.9% (51/74) of ATP-treated episodes in patients younger than 65.

Supplemental Tables

Supplemental Table 1. Procedure and defibrillation testing details

Characteristic	Subjects with an implant attempted (N = 787) (Mean ± SD or N, %)
Lead placement success	781 (99.2%)
Completing procedure with a system in place	758 (96.3%)
TYRX antibacterial envelope used	222/786 (28.2%)
Mean time from first incision to final closure (minutes)	81.0 ± 38.1 (N=690)
Lead repositioning performed for sensing	285/770 (37.0%)
Re-tunneling performed for sensing	123/770 (16.0%)
Type of sensing issue	
Atrial oversensing	22/123 (17.9%)
Suboptimal R-wave	62/123 (50.4%)
Both	36/123 (29.3%)
Unknown	3/123 (2.4%)
Mean number of tunneling attempts (all subjects, including initial tunneling)	1.3 ± 0.8 (N=767)
Mean number of re-tunneling attempts (subjects who re-tunneled, excluding initial tunneling)	1.7 ± 1.2
P-waves present at implant (via analyzer)	242/758 (31.9%)
P-waves present at implant (via device)	206/754 (27.3%)

Mean R-wave amplitude ± SD (Ring 1 to Ring 2) (mV)*	2.9 ± 1.6 (N=734)
Met P-wave and R-wave recommendations†	723/740 (97.7%)
Defibrillation testing performed	716 (91.0%)
Reasons for no defibrillation test‡	
Precluding condition, per investigator	17/52 (32.7%)
Defibrillation test planned for a later date	8/52 (15.4%)
Unable to induce a sustained ventricular arrythmia	15/52 (28.8%)
Other	13/52 (25.0%)
Reason not given	3/52 (5.8%)
Troubleshooting performed to improve defibrillation outcome	40/716 (5.6%)
Troubleshooting actions taken‡	
Adjust can position	25/40 (62.5%)
Check for/resolve air in pocket	5/40 (12.5%)
Check for/resolve air in tunnel	7/40 (17.5%)
Check for/resolve pneumothorax	2/40 (5.0%)
Check for/resolve high impedance values	3/40 (7.5%)
Reverse Shock Polarity	5/40 (12.5%)
Other	13/40 (32.5%)
Mean shock impedance (Ohms)	69.5 ± 19.5 (N=489)
Median (25th – 75th percentile)	67.0 (59 – 77)
Defibrillation testing success when known	705/713 (98.9%)
Mean successful defibrillation energy (J)	31.2 ± 4.4 (N=705)
Minimum energy tested among those with known outcome	

≤ 30 J	652/713 (91.4%)
> 30 J and < 40 J	13/713 (1.8%)
40 J	42/713 (5.9%)
Unknown	6/713 (0.8%)
Successful defibrillation test[§]	
≤ 30 J	572/652 (87.7%)
> 30 J	75/652 (11.5%)
Failure	5/652 (0.8%)

J: joules; mV: millivolts; SD: standard deviation

[*]Among successful implants

[†]R-wave amplitude (any vector) of ≥ 1.0 mV and, via analyzer or device, either no P-waves present or sensitivity at which P-waves no longer sensed ≤ 0.2 mV or sensitivity at which P-waves no longer sensed ≤ 0.3 mV and ratio of R-wave amplitude to sensitivity ≥ 10

[‡]Not mutually exclusive

[§]Among those with minimum energy tested of ≤ 30 J and with a known outcome

Supplemental Table 2. Device programming as of most recent interrogation

Programmed parameter	Patients with device follow-up data (N=706) (N, %)
VF therapy “on”	702 (99.4%)
ATP programmed “on”	623 (88.2%)
VT zone ATP programming “on”	139 (19.7%)
FVT via VF zone ATP programming “on”	598 (84.7%)
VF zone number of beats to detect	
≤ 18 of 24	10 (1.4%)
24 of 32	7 (1.0%)
30 of 40	602 (85.8%)
45 of 60	60 (8.6%)
≥60 of 80	23 (3.3%)
VT zone number of beats to detect	
16	21 (15.1%)
20	2 (1.4%)
24	12 (8.6%)
28	11 (7.9%)
32	24 (17.3%)
36	13 (9.4%)
40	36 (25.9%)
≥44	20 (14.4%)
VF zone interval (ms)	
280	108 (15.4%)
290	33 (4.7%)
300	333 (47.4%)

310	12 (1.7%)
320	199 (28.4%)
≥330	17 (2.4%)
FVT via VF zone interval (ms)	
200	24 (4.0%)
210	2 (0.3%)
220	8 (1.3%)
230	13 (2.2%)
240	244 (40.8%)
250	130 (21.7%)
260	76 (12.7%)
270	60 (10.0%)
≥280	41 (6.9%)
VT zone interval (ms)	
310	4 (2.9%)
320	35 (25.2%)
330	34 (24.5%)
340	22 (15.8%)
350	12 (8.6%)
360	19 (13.7%)
≥370	13 (9.3%)

ATP: antitachycardia pacing; ms: milliseconds; FVT: fast ventricular tachycardia; VT/VF: ventricular tachycardia/fibrillation

Supplemental Table 3. Reasons for inappropriate shock

Reason	Inappropriately shocked episodes (N, %)
Non-cardiac oversensing*	30 (40.5%)
P-wave oversensing	23 (31.1%)
AF/ Atrial Flutter	12 (16.2%)
Other SVT/Sinus Tachycardia	7 (9.5%)
Non-sustained VT	1 (1.4%)
T-wave oversensing	1 (1.4%)

Proportions calculated out of 74 inappropriately shocked episodes.

AF: atrial fibrillation; SVT: supraventricular tachycardia; VT: ventricular tachycardia

Mean device follow-up duration for the 706 patients with device data was 12.9 ± 5.6 months

*Non-cardiac oversensing included lead noise (27 episodes, 36.5%) and electromagnetic interference (3 episodes, 4.1%)

Supplemental Table 4. Listing of chronic system-related major complications (primary endpoint) through one year

Chronic major system-related complications	Number of events (number, % of subjects with events)
Inappropriate shock resulting in hospitalization or system revision	9 (9, 1.1%)
Lead dislodgement	5 (5, 0.7%)
Inappropriate ATP due to fast AF*	2 (2, 0.3%)
Pocket revision to relieve pain	2 (2, 0.3%)
Impaired wound healing	1 (1, 0.1%)
Pocket erosion	1 (1, 0.1%)
Oversensing	1 (1, 0.1%)
High lead impedance	1 (1, 0.1%)
**Total**	**22 (22, 2.9%)**

AF: atrial fibrillation; ATP: antitachycardia pacing

*In both cases the patient was hospitalized following inappropriate ATP to receive an ablation procedure for AF

Supplemental Table 5. Listing of system revisions that occurred through one year grouped by occurrence post-implantation

Reason for system modification	Number of events (number, % of subjects with events)		
	≤ 30 days	> 30 days	Total
**System explant**			
Infection	1 (1, 0.1%)	8 (8, 1.0%)	9 (9, 1.1%)
Lead dislodgement	5 (5, 0.7%)	0 (0, 0%)	5 (5, 0.7%)
Heart Transplant	0 (0, 0%)	3 (3, 0.4%)	3 (3, 0.4%)
Inappropriate shock	0 (0, 0%)	3 (3, 0.4%)	3 (3, 0.4%)
Failed defibrillation test	2 (2, 0.3%)	0 (0, 0%)	2 (2, 0.3%)
Sub-optimal R-wave	1 (1, 0.1%)	0 (0, 0%)	1 (1, 0.1%)
System upgrade	0 (0, 0%)	1 (1, 0.1%)	1 (1, 0.1%)
Pocket erosion	0 (0, 0%)	1 (1, 0.1%)	1 (1, 0.1%)
**Lead repositioning**			
Lead dislodgement	2 (2, 0.3%)	0 (0, 0%)	2 (2, 0.3%)
Inappropriate shock	0 (0, 0%)	1 (1, 0.1%)	1 (1, 0.1%)
Sub-optimal lead placement	1 (1, 0.1%)	0 (0, 0%)	1 (1, 0.1%)
Failed defibrillation test	1 (1, 0.1%)	0 (0, 0%)	1 (1, 0.1%)
Pocket site pain*	0 (0, 0%)	1 (1, 0.1%)	1 (1, 0.1%)
**Generator repositioning**			
Failed defibrillation test	2 (2, 0.3%)	0 (0, 0%)	2 (2, 0.3%)
Device rotation	1 (1, 0.1%)	0 (0, 0%)	1 (1, 0.1%)
Infection	0 (0, 0%)	1 (1, 0.1%)	1 (1, 0.1%)

Pocket site pain*	0 (0, 0%)	1 (1, 0.1%)	1 (1, 0.1%)
**Lead explant and replacement with new lead**			
Lead dislodgement	0 (0, 0%)	7 (7, 0.9%)	7 (7, 0.9%)
Inappropriate shock	1 (1, 0.1%)	0 (0, 0%)	1 (1, 0.1%)
**Total**	**17 (17, 2.2%)**	**27 (26, 3.4%)**	**44 (43, 5.7%)**

Proportions were calculated out of patients with an implant attempt (N=787). Timing is indicative of when the system modification was performed post-implantation, which did not always occur on the same day as the event that led to the revision. *One patient had both generator and lead repositioning performed at the same time for the same event (pocket site pain).

Supplemental Table 6. Deaths through one year

Cause of death	(N, %)[*]
Acute kidney injury	1 (0.1%)
Benign oesophageal neoplasm	1 (0.1%)
Cardiac arrest[*]	2 (0.3%)
Cardiogenic shock	1 (0.1%)
Hypoxic-ischemic encephalopathy[†]	1 (0.1%)
Malignant neoplasm of unknown primary site	1 (0.1%)
Multiple organ dysfunction syndrome	1 (0.1%)
Myocardial infarction	1 (0.1%)
Sepsis[‡]	1 (0.1%)
Septic shock[§]	3 (0.4%)
Unknown cause	3 (0.4%)
**Total deaths**	**16 (2.0%)**

Proportions calculated out of those with an implant attempt (N=787)

[*]Both patients had the EV-ICD system turned off prior to expiration from cardiac arrest. In one case EV-ICD was turned off for end-of-life reasons after the patient's health deteriorated and a do not resuscitate order; VT/VF involvement was not suspected in this death. In the second case, EV-ICD was turned off due to inappropriate shock and the patient experienced a VT/VF episode and was not compliant with wearing a prescribed LifeVest at the time.

[†]Three days prior to expiration the patient experienced a VT/VF storm treated with both EV-ICD and external shocks, with an EV-ICD shock terminating the rhythm.

[‡]Source of infection not known, but no evidence that the infection reached any component of the system and patient was undergoing hemodialysis for renal failure.

[§]One septic shock was secondary to gastroenteritis, the second was due to anastomotic leak following large bowel resection, and the third originated at the aortic valve in a patient with a history of endocarditis. There was no evidence that the infections involved or reached any part of the system.

Supplemental Table 7. Baseline characteristics and medical history grouped by age

Characteristic	Age < 65 (N = 651) (Mean ± SD or N, %)	Age ≥ 65 (N = 136) (Mean ± SD or N, %)	P-value
Age (years)			< 0.0001
Mean ± SD	44.6 ± 12.6	70.4 ± 4.5	
Minimum - Maximum	18 - 64	65 - 84	
Sex			0.022
Female	198 (30.4%)	28 (20.6%)	
Male	453 (69.6%)	108 (79.4%)	
ICD Indication			0.37
Primary prevention	434 (66.7%)	85 (62.5%)	
Secondary prevention	217 (33.3%)	51 (37.5%)	
NYHA Class			0.029
I	104 (16.0%)	17 (12.5%)	
II	148 (22.7%)	49 (36.0%)	
III	42 (6.5%)	9 (6.6%)	
IV	6 (0.9%)	1 (0.7%)	

Characteristic	Age < 65 (N = 651) (Mean ± SD or N, %)	Age ≥ 65 (N = 136) (Mean ± SD or N, %)	P-value
Subject does not have heart failure / Not available	351 (53.9%)	60 (44.1%)	
BMI (kg/m^2)			0.52
Mean ± SD	27.4 ± 6.0 (N=645)	27.7 ± 4.9 (N=135)	
LV Ejection Fraction (%)			0.007
Mean ± SD	45.5 ± 16.0 (N=585)	41.2 ± 15.9 (N=124)	
Cardiac arrest	143 (22.0%)	23 (16.9%)	0.21
Cardiomyopathy	424 (65.1%)	104 (76.5%)	0.012
Ischemic	114 (17.5%)	52 (38.2%)	< 0.0001
Non-ischemic	191 (29.3%)	32 (23.5%)	0.21
Hypertrophic	124 (19.0%)	22 (16.2%)	0.47
Coronary artery disease	105 (16.1%)	59 (43.4%)	< 0.0001
Hypertension	171 (26.3%)	71 (52.2%)	< 0.0001
Primary/idiopathic electrical disease	125 (19.2%)	10 (7.4%)	< 0.001
Arrhythmogenic RV dysplasia	28 (4.3%)	1 (0.7%)	0.044

Characteristic	Age < 65 (N = 651) (Mean ± SD or N, %)	Age ≥ 65 (N = 136) (Mean ± SD or N, %)	P-value
Brugada syndrome	32 (4.9%)	2 (1.5%)	0.10
Long Q/T syndrome	22 (3.4%)	3 (2.2%)	0.60
Unknown type	18 (2.8%)	2 (1.5%)	0.55
Other	26 (4.0%)	2 (1.5%)	0.20
Atrial fibrillation	70 (10.8%)	39 (28.7%)	< 0.0001
Ventricular arrhythmias	340 (52.2%)	74 (54.4%)	0.71
Ventricular fibrillation	113 (17.4%)	17 (12.5%)	0.20
Diabetes	93 (14.3%)	48 (35.3%)	< 0.0001
Renal dysfunction	45 (6.9%)	26 (19.1%)	< 0.0001

BMI: body mass index; ICD: implantable cardioverter-defibrillator; kg: kilograms; m: meters; NYHA: New York Heart Association; RV/LV: right/left ventricular; SD: standard deviation

Supplemental Table 8. Procedure and defibrillation testing details by age

Characteristic	Age < 65 (N = 651) (Mean ± SD or N, %)	Age ≥ 65 (N = 136) (Mean ± SD or N, %)	P-value
Lead placement success	645 (99.1%)	136 (100.0%)	0.60
Completed procedure with system in place	631 (96.9%)	127 (93.4%)	0.074
Mean time from first incision to final closure (minutes)	81.7 ± 39.3 (N=570)	77.8 ± 31.3 (N=120)	0.25
Lead repositioning performed for sensing	226/639 (35.4%)	59/131 (45.0%)	0.047
Re-tunneling performed for sensing	95/639 (14.9%)	28/131 (21.4%)	0.068
Type of sensing issue			0.84
Atrial oversensing	18/95 (18.9%)	4/28 (14.3%)	
Suboptimal R-wave	46/95 (48.4%)	16/28 (57.1%)	
Both	28/95 (29.5%)	8/28 (28.6%)	
Unknown	3/95 (3.2%)	0 (0.0%)	
Number of tunneling attempts (all subjects, including initial tunneling)	1.3 ± 0.8 (N=637)	1.4 ± 0.8 (N=130)	0.18
Mean number of re-tunneling attempts (subjects who re-tunneled, excluding initial tunneling)	1.7 ± 1.2	1.7 ± 1.0	0.92
P-waves present at implant (via analyzer)	204/629 (32.4%)	38/129 (29.5%)	0.54
P-waves present at implant (via device)	175/627 (27.9%)	31/127 (24.4%)	0.45
Mean R-wave amplitude (Ring 1 to Ring 2) (mV)*	3.0 ± 1.7 (N=612)	2.3 ± 1.1 (N=122)	<0.001

Met P-wave and R-wave recommendations†	603/617 (97.7%)	120/123 (97.6%)	1.00
Defibrillation testing performed	604 (92.8%)	112 (82.4%)	<0.001
Reasons for no defibrillation test‡			
Precluding condition, per investigator	14/33 (42.4%)	3/19 (15.8%)	
Defibrillation test planned for a later date	5/33 (15.2%)	3/19 (15.8%)	
Unable to induce a sustained ventricular arrythmia	7/33 (21.2%)	8/19 (42.1%)	
Other	9/33 (27.3%)	4/19 (21.1%)	
Reason not given	2/33 (6.1%)	1/19 (5.3%)	
Troubleshooting performed to improve defibrillation outcome	31/604 (5.1%)	9/112 (8.0%)	0.16
Troubleshooting actions taken‡			
Adjust can position	20/31 (64.5%)	5/9 (55.6%)	
Check for/resolve air in pocket	4/31 (12.9%)	1/9 (11.1%)	
Check for/resolve air in tunnel	4/31 (12.9%)	3/9 (33.3%)	
Check for/resolve pneumothorax	2/31 (6.5%)	0 (0.0%)	
Check for/resolve high impedance values	2/31 (6.5%)	1/9 (11.1%)	
Reverse Shock Polarity	2/31 (6.5%)	3/9 (33.3%)	
Other	12/31 (38.7%)	1/9 (11.1%)	
Mean shock impedance (Ohms)	69.1 ± 19.9 (N=415)	72.2 ± 17.1 (N=74)	0.21
Median (25th – 75th percentile)	67.0 (59 – 76)	71.0 (61 – 81)	

Defibrillation testing success when known	593/601 (98.7%)	112/112 (100.0%)	0.62
Mean minimum successful defibrillation energy (J)	31.2 ± 4.3 (N=593)	31.0 ± 4.9 (N=112)	0.71
Minimum energy tested among those with known outcome			0.53
≤ 30 J	551/601 (91.7%)	101/112 (90.2%)	
> 30 J and < 40 J	10/601 (1.7%)	3/112 (2.7%)	
40 J	34/601 (5.7%)	8/112 (7.1%)	
Unknown	6/601 (1.0%)	0 (0.0%)	
Successful defibrillation test[§]			1.00
≤ 30 J	482/551 (87.5%)	90/101 (89.1%)	
> 30 J	64/551 (11.6%)	11/101 (10.9%)	
Failure	5/551 (0.9%)	0 (0.0%)	

J: joules; mV: millivolts; SD: standard deviation

[*]Among successful implants

[†]R-wave amplitude (any vector) of ≥ 1.0 mV and, via analyzer or device, either no P-waves present or sensitivity at which P-waves no longer sensed ≤ 0.2 mV or sensitivity at which P-waves no longer sensed ≤ 0.3 mV and ratio of R-wave amplitude to sensitivity ≥ 10

[‡]Not mutually exclusive

[§]Among those with minimum energy tested of ≤ 30 J and with a known outcome

Supplemental Figures

Supplemental Figure 1. Freedom from chronic, system-related major complications (primary endpoint) through one year

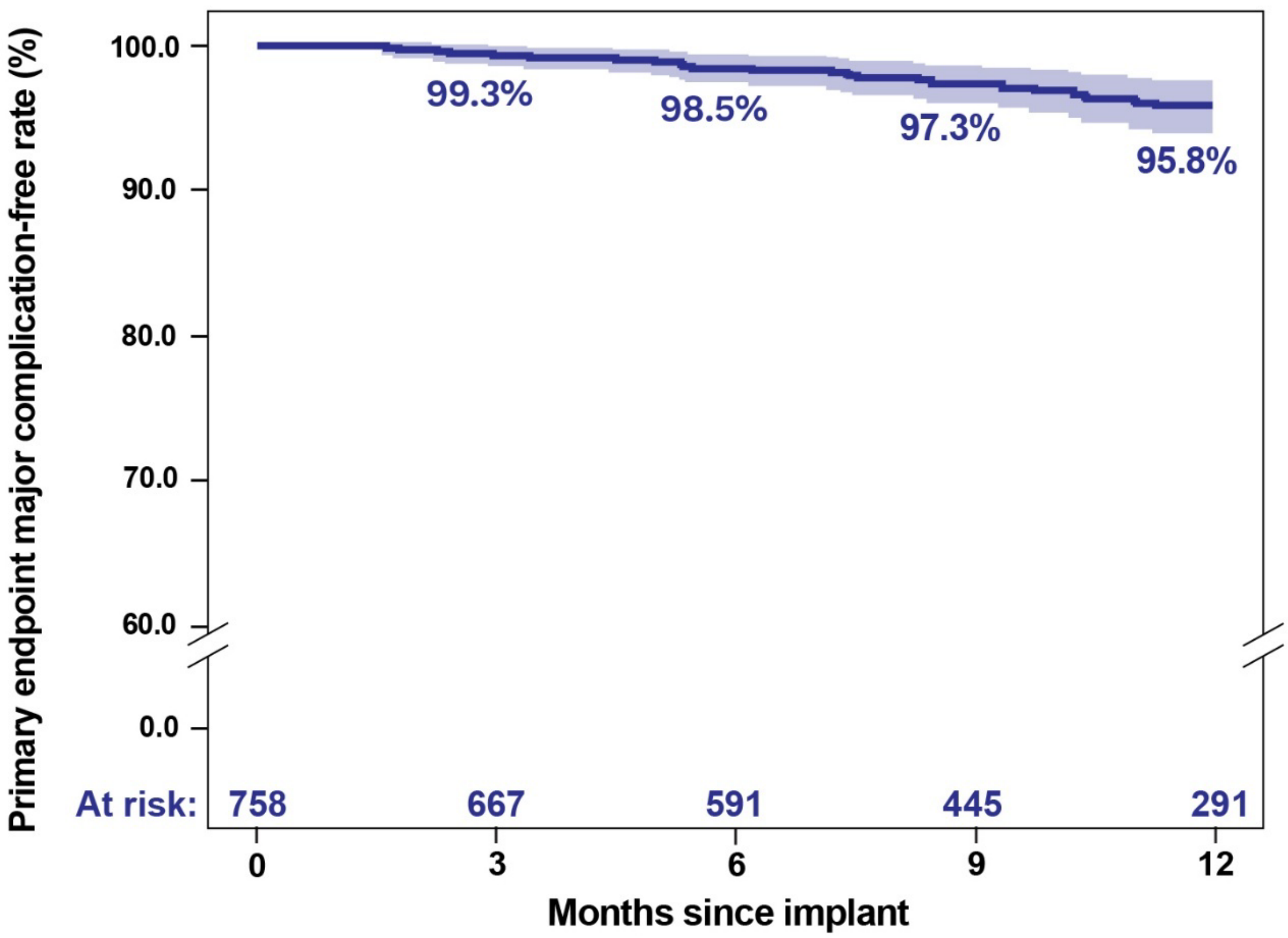

Legend: The primary endpoint of Enlighten is freedom from chronic (> 30 days) system-related major complications (excluding infections) through five years in those with a successful procedure (n=758). Kaplan-Meier curve in blue gives an update of the primary endpoint at one year with the number of patients at risk for each timepoint shown at the bottom in blue. Shaded blue region represents the 95% confidence intervals (CI).

Supplemental Figure 2. Kaplan-Meier freedom from all major system- and/or procedure-related complications by age group

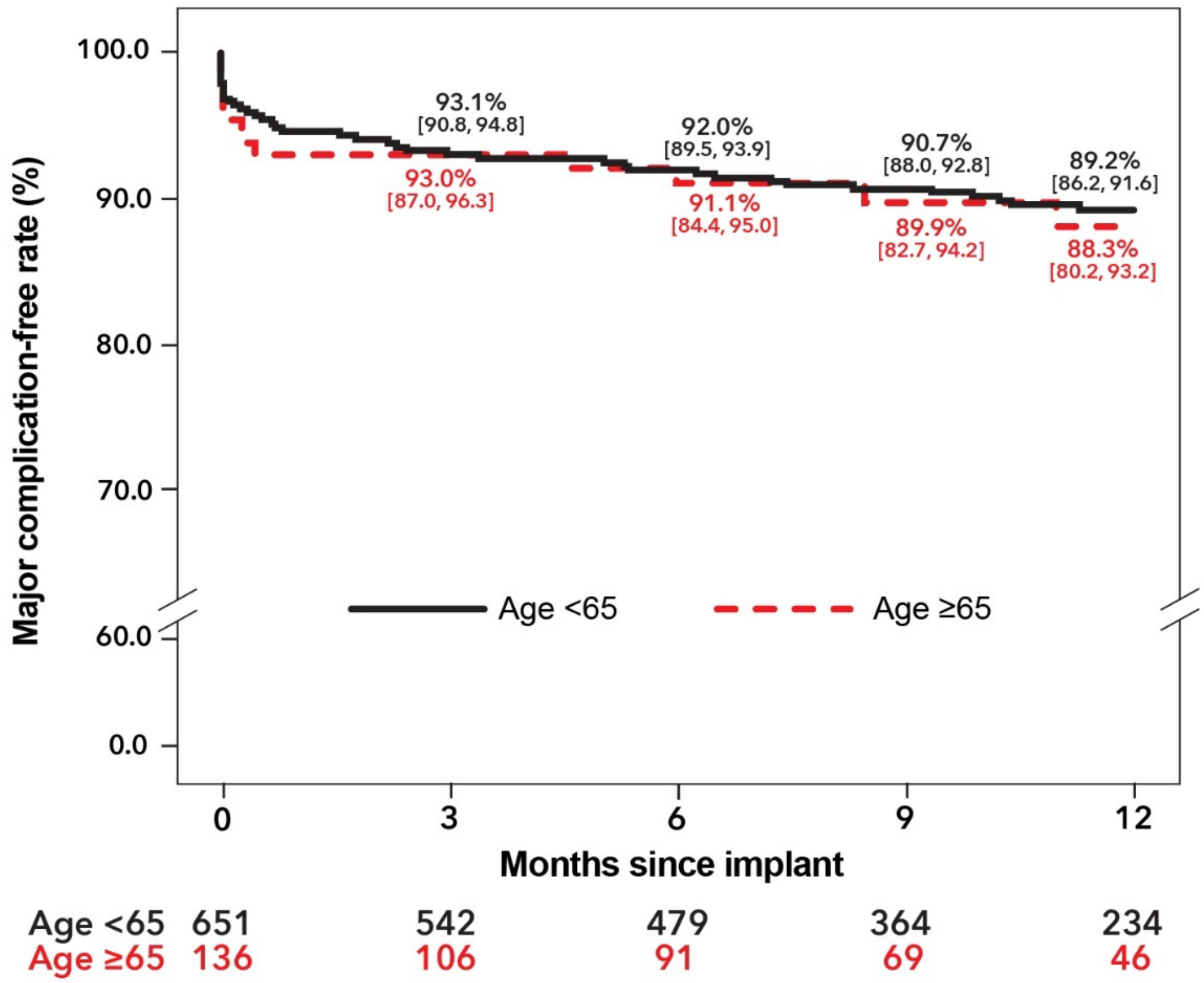

Legend: Kaplan-Meier estimated freedom from all system- and/or procedure-related major complications through one year for patients with an implant attempt (N=787). Patients younger than 65 depicted in black and patients 65 or older in red. The number at risk for each timepoint is shown at the bottom in corresponding colors. Numbers in brackets on the graph represent the 95% confidence intervals (CI).

Supplemental Figure 3. Rate of appropriate therapy by age group

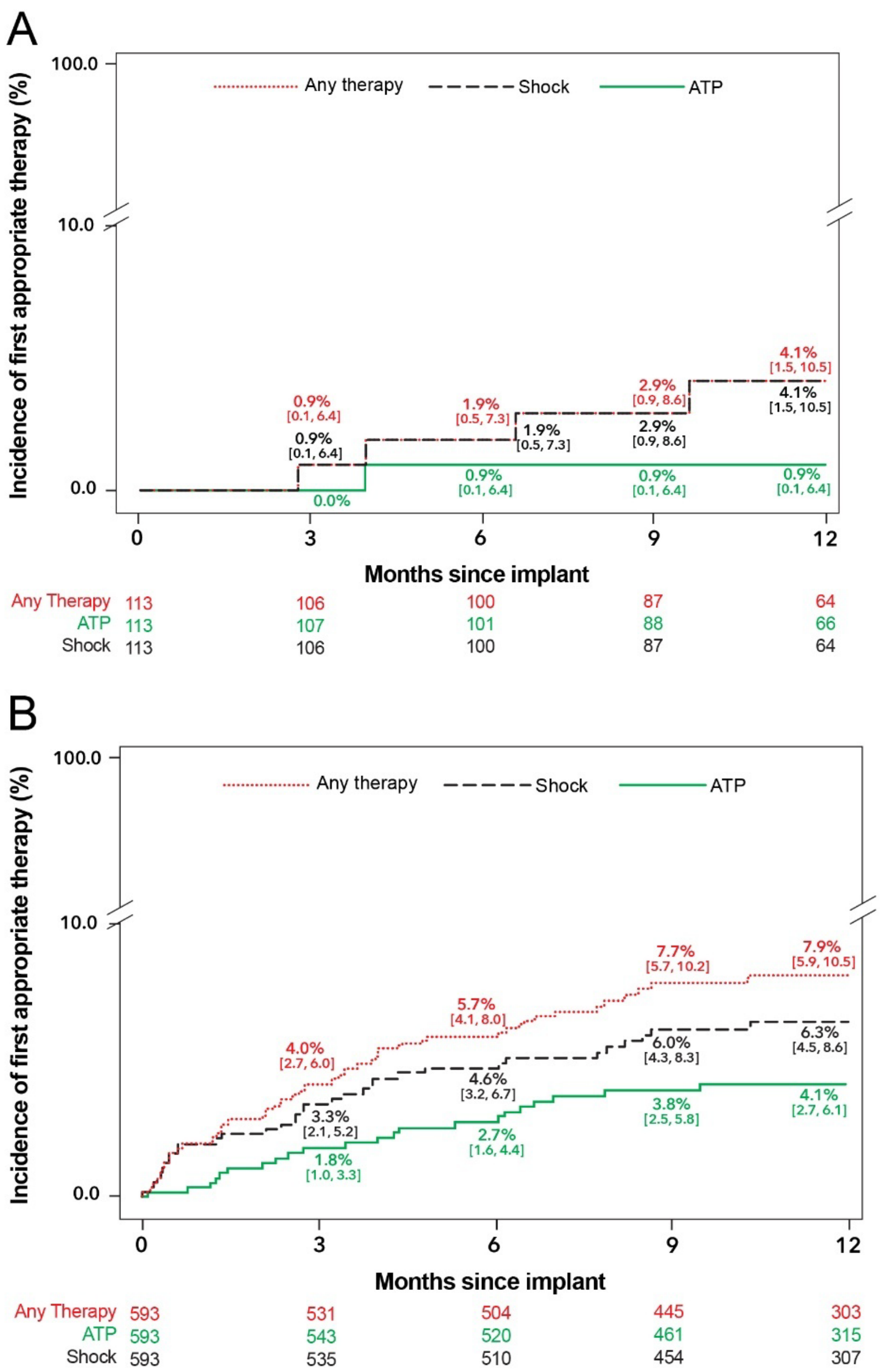

Legend: Kaplan-Meier estimated rates of appropriate therapy for patients ≥ 65 years old **(A)** and patients younger than 65 **(B)**. Both graphs are through one year; red line depicts any therapy (Shock, ATP, or both); green line depicts ATP therapy; black line depicts shock therapy. The

number at risk for each timepoint is shown at the bottom of each graph in corresponding colors. Numbers in brackets on the graphs represent the 95% confidence intervals (CI).

Supplemental Figure 4. Rate of inappropriate therapy by age group

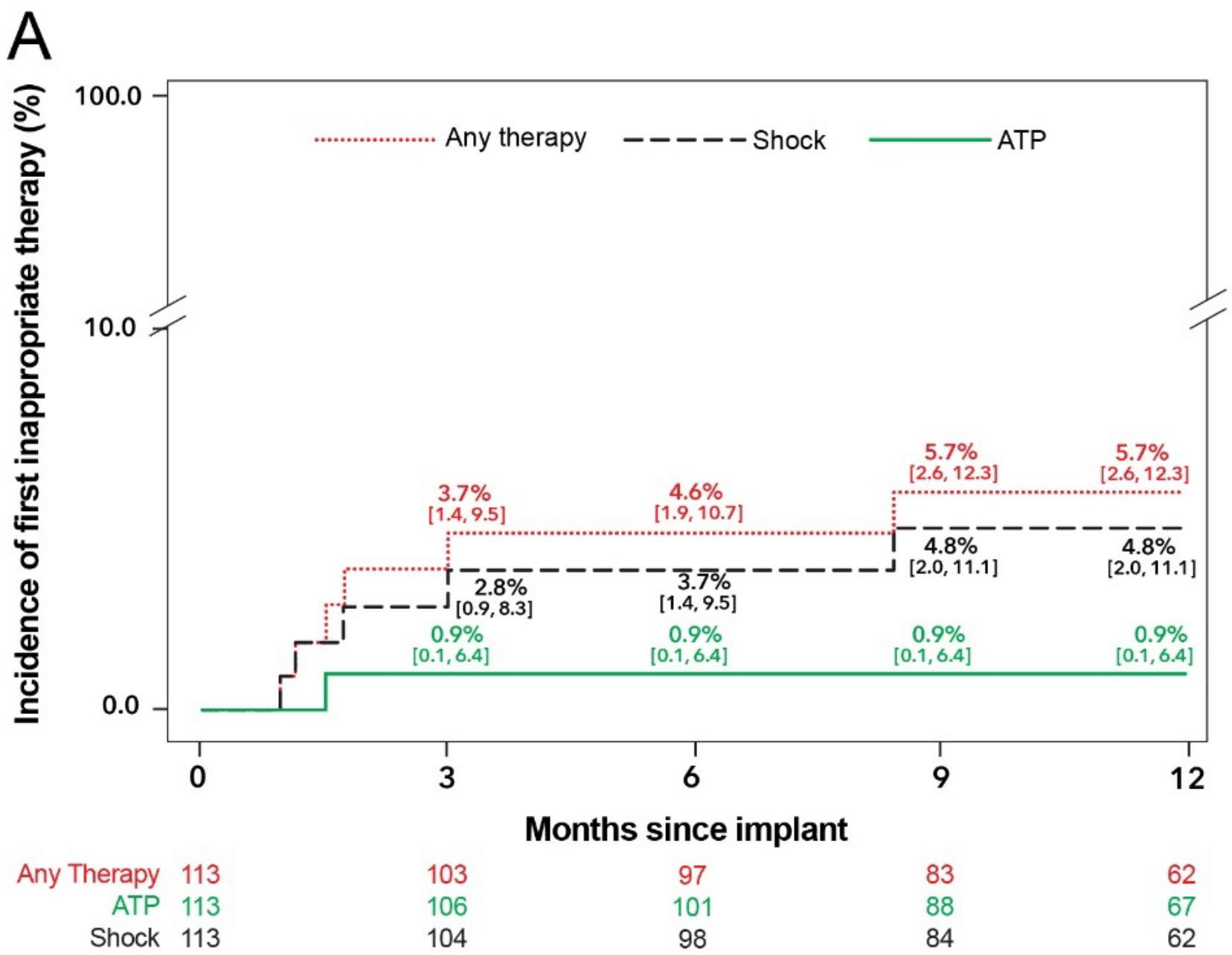

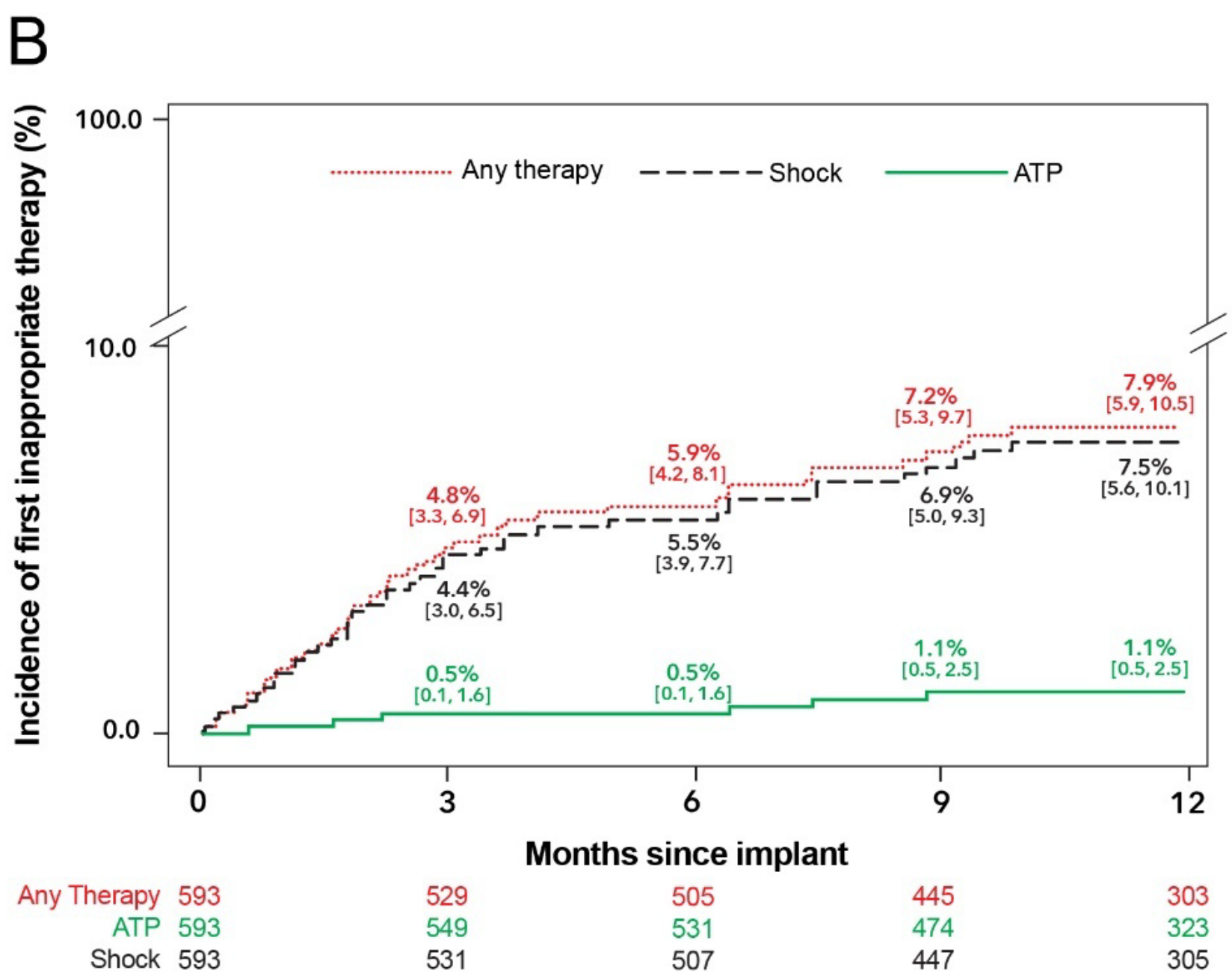

Legend: Kaplan-Meier estimated rates of inappropriate therapy for patients ≥ 65 years old **(A)** and patients younger than 65 **(B)**. Both graphs are through one year; red line depicts any therapy (Shock, ATP, or both); green line depicts ATP therapy; black line depicts shock therapy. The number at risk for each timepoint is shown at the bottom of each graph in corresponding colors. Numbers in brackets on the graphs represent the 95% confidence intervals (CI).
